# La praxis de la atención en salud mental y la manifestación del estigma: un estudio etnográfico en centros de salud mental de la Región del Biobío, Chile

**DOI:** 10.18294/sc.2025.5631

**Published:** 2025-10-08

**Authors:** Romina Jara-Ogeda, Pamela Grandón Fernández, Daniela Leyton Legües

**Affiliations:** 1 Magíster en Psicología. Jefe técnica, programa Familias de Acogida Especializada, FAE Ayün, Corporación de Apoyo a la Niñez y Juventud en Riesgo Social Llequén, Los Ángeles, Chile. psromina.jarao@gmail.com Corporación de Apoyo a la Niñez y Juventud en Riesgo Social Llequén programa Familias de Acogida Especializada, FAE Ayün Corporación de Apoyo a la Niñez y Juventud en Riesgo Social Llequén Los Ángeles Chile psromina.jarao@gmail.com; 2 Doctora en Psicología. Profesora titular, Departamento de Psicología, Universidad de Concepción, Concepción, Chile. pgrandon@udec.cl Universidad de Concepción Departamento de Psicología Universidad de Concepción Concepción Chile pgrandon@udec.cl; 3 Autora de correspondencia: Doctora en Ciencias Humanas. Profesora asociada, Facultad de Ciencias Sociales, Universidad de Concepción, Concepción, Chile. danielaleyton@udec.cl Universidad de Concepción Facultad de Ciencias Sociales Universidad de Concepción Concepción Chile danielaleyton@udec.cl

**Keywords:** Salud Mental, Trastorno Mental, Estigma Social, Sistemas de Salud, Etnografía, Chile

## Abstract

En este artículo nos preguntamos cómo la praxis en salud mental reproduce el estigma hacia usuarios de los centros de salud mental en la Región del Biobío en Chile, partiendo del espacio relacional de las interacciones, donde el estigma se expresa y sostiene. Desde un diseño de investigación cualitativa con enfoque etnográfico, de junio a septiembre de 2018, se realizó observación participante en contextos formales e informales de los dispositivos de salud, entrevistas de tipo etnográfica y entrevistas semiestructuradas. Identificamos una constante tensión entre el modelo comunitario en salud mental, promovidas por las políticas de salud pública, y las formas biomédicas en la atención de usuarios con diagnóstico psiquiátrico, que impactan en la práctica cotidiana de atención del personal de salud y, por ende, en el tipo de intervención que los usuarios reciben en la interacción y participación en los dispositivos de salud. Dichas tensiones se manifiestan en al menos dos ámbitos: por un lado, en la preponderancia del modelo biomédico frente a otras prácticas médicas, impactando y constriñendo el ejercicio de su rol profesional; y, por otro, se expresa en las condiciones del trabajo que deben desempeñar los profesionales en salud mental, lo que los conduce a experimentar agotamiento. Sostenemos que ambos ámbitos, de manera interrelacionada, impactan en las trayectorias de atención en salud mental e inciden en la reproducción del estigma hacia personas que padecen enfermedades de salud mental.

## Introducción

Las personas con diagnóstico psiquiátrico son frecuentemente objeto de estigma lo que tiene múltiples y negativas consecuencias para los afectados^1^. El estigma alude al proceso de etiquetamiento, pérdida de estatus, y discriminación hacia personas que presentan una característica devaluada socialmente^2^. Para Goffman^3^, los procesos de construcción social son fundamentales para comprender el estigma. Este se configura a partir de atributos negativos que desvalorizan al individuo, reduciéndolo a estos y relegando a un segundo plano las demás características que lo definen, lo que permite que el estigma llegue a ser percibido como el rasgo identitario central de la persona. Este proceso de internalización del estigma, acompañado del comportamiento que se ajusta al rol de paciente, ha sido denominado por Goffman como la “carrera moral del enfermo”. Este concepto revela cómo, más allá del tratamiento clínico, el personal de salud contribuye -consciente o inconscientemente- a la construcción social del estigma y a la imposición de un rol restringido a las personas con diagnóstico psiquiátrico

Cuando las personas son definidas principalmente por su diagnóstico y se validan representaciones asociadas a la cronicidad, la incapacidad o la peligrosidad en la práctica cotidiana de la atención sanitaria, las y los profesionales de salud mental del nivel secundario de atención suelen reproducir el estigma hacia las personas con diagnóstico psiquiátrico^4^. Así, se materializan las concepciones de los miembros de los equipos de salud mental sobre los usuarios, las formas en que entienden lo que les sucede y cómo abordan sus situaciones, es decir, el modelo de atención que poseen y aplican en su práctica^5^.

En Chile, la política de salud mental está basada en el modelo comunitario de atención en salud mental, entendido como un conjunto de principios y prácticas necesarias para promover la salud mental de grupos y comunidades a través de, principalmente, la organización y estructura de los servicios sanitarios^6^. En el país, el nivel secundario de salud entrega cuidados a las personas con diagnóstico de trastornos mentales de mayor complejidad en las Unidades de Psiquiatría Ambulatorias, los Centros Comunitarios de Salud Mental (COSAM) y los hospitales de día^7^. Estos dispositivos de salud son del sector público que atiende a cerca del 80% de la población del país a través del Fondo Nacional de Salud (FONASA), que es un seguro estatal de salud^8^. El FONASA concentra gran parte de la población adulta mayor y vulnerable. Su cobertura es mayor en mujeres (81,2%) que en hombres (76,6%), y alcanza a más del 90% en los dos primeros quintiles de ingresos, mientras que en el quintil más alto se reduce al 37,9%^9^. Por recomendaciones de la Organización Mundial de la Salud (OMS) estos servicios deben estar en el territorio en el que residen las personas usuarias y sus comunidades, integrados a los servicios de salud general y contar con equipos multidisciplinarios. Desde esta perspectiva las y los usuarios son entendidos como sujetos integrales y autónomos, y lo que les sucede se deriva de aspectos no solo biológicos, sino que también psicosociales^10^. Las personas diagnosticadas con trastorno mental, en esta visión, habitan en un contexto social vulnerable y presentan distintas capacidades cognitivas, emocionales y relacionales que le hacen difícil afrontar las dificultades^11^. Este enfoque disminuiría la intolerancia y prejuicio y aumentaría la empatía y deseo de apoyo hacia las personas con diagnóstico de trastorno mental^12^.

No obstante, en la praxis sigue primando el enfoque o modelo biomédico de atención. De acuerdo con Menéndez^13^^,^^14^, la biomedicina se ha conformado como el modelo médico hegemónico (MMH) frente a otras formas de abordar los procesos de salud, enfermedad y atención, producto de conformaciones históricas, socioeconómicas y políticas particulares acaecidos en las sociedades occidentales. El MMH se caracteriza, entre otros aspectos, por una relación jerárquica entre médico-paciente, por la individualización y el reduccionismo del sujeto al desvincularlo de su contexto sociohistórico, y por un biologicismo exacerbado^13^^,^^14^. En el campo de la salud mental, el MMH aborda los llamados trastornos mentales, desde una lógica biomédica en la que estos se explican por una disfunción del cerebro, es decir, son enfermedades basadas en anormalidades genéticas y neurobiológicas y, por consiguiente, tienen la misma naturaleza que las enfermedades físicas; de ahí que su tratamiento se fundamenta en un adecuado diagnóstico y prescripción farmacológica, para los que se buscan signos y síntomas de la patología^15^. De esta forma, el sufrimiento de las personas se individualiza y despoja de su contexto sociocultural. En este enfoque jerárquico, el médico, en este caso el psiquiatra es quien tiene el mayor estatus, seguido por el psicólogo^16^. Ambos profesionales “psi” están legitimados socialmente para hacer diagnóstico psicológico o psiquiátrico, mientras que la prescripción médica es realizada por el psiquiatra. En este modelo, la persona termina siendo monitoreada desde la presencia o ausencia de síntomas^17^ que son cotejados en el manual diagnóstico DSM-5. La relevancia del diagnóstico y la medicación en la atención es tal, que otros profesionales terminan enfatizando estos aspectos pese a tener otras miradas sobre los usuarios^16^. En el personal de salud, se ha encontrado que este modelo aumenta el deseo de distancia social, disminuye la empatía e interfiere en la relación terapéutica con el usuario de salud mental^18^, lo que puede generar actitudes negativas y deshumanización hacia estos^19^^,^^11^. 

Por tanto, los equipos de salud mental utilizan diversos modelos de atención, entre ellos, el modelo comunitario de atención en salud mental y el MMH, que se contradicen en su comprensión de los usuarios y la forma de abordar lo que les sucede. Al personal de salud mental se le solicita una comprensión integral del usuario desde el modelo comunitario de atención en salud mental, pero en la práctica se ven compelidos a funcionar bajo la lógica del MMH, lo que les genera tensión y termina tiñendo su experiencia de trabajo^20^^,^^21^. Consideramos entonces, que es importante tener en cuenta el impacto que tiene en los profesionales su trabajo en salud mental y estar atentos a sus necesidades, para evitar la afectación de su propia salud mental^22^.

Podemos destacar que, en Chile, aún es reducida la investigación sobre el estigma hacia los trastornos mentales entre el personal de salud. La mayoría de los estudios se han realizado con profesionales de atención primaria; estos presentan rechazo, desconfianza, minimizan los síntomas físicos de los usuarios y los ven como peligrosos^23^^,^^24^^,^^25^. Las pocas investigaciones con el personal especializado en salud mental muestran que también estigmatiza a los usuarios, pero lo hace desde interacciones que menoscaban y restringen sus derechos y posibilidades de participación en el tratamiento, lo que obstaculiza la recuperación^26^^,^^27^. Estos resultados son incipientes por lo que estimamos que se requiere mayor investigación para conocer cómo se presenta el estigma en este grupo de trabajadores, sobre todo teniendo presente que su reducción es parte del Plan Nacional de Salud Mental^7^.

### El ingreso etnográfico a centros de salud mental en la región del Biobío

La realización de una etnografía para llevar a cabo la investigación, la sustentamos en la necesidad de explorar la manifestación del estigma desde la perspectiva de los profesionales en su contexto habitual de trabajo, permitiéndonos describir las situaciones ocurridas con base en la observación de las acciones de los propios protagonistas^28^. Asimismo, buscamos indagar en las ideas, creencias, significados, conocimientos y prácticas compartidas por un grupo particular^29^, en este caso, desplegadas en el contexto laboral cotidiano de los profesionales de la salud que trabajan en centros públicos de atención secundaria en salud mental de la región del Biobío. A partir del enfoque etnográfico, desarrollamos una descripción contextualizada sustentada en la observación participante^28^, aproximándonos a las interacciones sociales entre los profesionales. Los datos etnográficos surgen a partir de nuestro análisis e interpretación como equipo de investigadoras, respecto de la información producida mediante observaciones, entrevistas o conversaciones etnográficas y entrevistas semiestructuradas^29^. 

El trabajo de campo etnográfico se realizó en dos de los tres Centros Comunitarios de Atención en Salud Mental (COSAM) y el único hospital de día situados en la Región del Biobío en el centro-sur de Chile. La elección se debió a que son centros de referencia para el tratamiento de personas diagnosticadas con trastornos mentales, y los tres lugares mencionados accedieron a participar en el estudio. El trabajo de campo se llevó a cabo durante tres meses, desde el 11 de junio al 11 de septiembre del año 2018, distribuyendo equitativamente entre los tres centros de salud el tiempo semanal de la observación participante. 

Como equipo de investigadoras, coincidimos respecto a las formas de producción de conocimiento enfocadas desde el paradigma constructivista y crítico, que sostienen que las realidades son construidas localmente y en contexto, sin negar la existencia de aspectos estructurales que condicionan dicha realidad. Las interacciones entre las investigadoras y los participantes ocurren en distintos niveles de colaboración lo que nos permite comprender el fenómeno de estudio de manera co-creada, sin olvidar, de forma coincidente con las perspectivas críticas, que los hallazgos se encuentran mediados por valores, es decir, que nuestra posición e historia como sujetos afecta a todo el proceso de investigación^30^. En ese sentido, destacamos que el acceso al campo se vio facilitado por la formación profesional en psicología de la autora principal, quien había realizado previamente una pasantía profesional en un centro de salud aledaño, lo que ayudó a que los profesionales accedieran a participar de forma voluntaria y accedieran a la observación de sus interacciones cotidianas.

Las técnicas de investigación utilizadas para producir la etnografía correspondieron a la observación participante durante tres meses, entrevistas etnográficas en contextos informales de interacción con profesionales y entrevistas semi-estructuradas. La observación participante se efectuó en los espacios de interacción entre los profesionales; y entre profesionales y las personas que acuden a buscar atención. La participación se dió en espacios formales, tales como reuniones clínicas de cada equipo, reuniones de ingreso en hospital de día, actividades grupales y visitas domiciliarias; como también, en situaciones cotidianas de tipo informal como los horarios de colación de los profesionales. Las observaciones se complementaron con las entrevistas etnográficas, comprendidas como una conversación entre investigadora y participantes que ocurre en el contexto de la observación de manera casual, sin estar previamente definidas, pero guiadas por los objetivos de la investigación^31^. Estas se realizaron durante todo el trabajo de campo, buscando abrir e identificar temáticas emergentes, así como también, profundizar en temas que surgieron durante las observaciones. 

Las entrevistas semiestructuradas se realizaron con la finalidad de profundizar en los temas emergentes del trabajo de campo etnográfico, tales como: experiencia de trabajo en salud mental; percepción sobre las instancias de reunión clínica; apreciaciones personales sobre funcionamiento de la farmacia de salud mental y prescripción de fármacos como práctica en salud; conceptualización y abordaje de “casos complejos”; y, por último, descripción y percepción sobre los talleres ofrecidos e instancias de visitas domiciliarias. Se llevaron a cabo cinco entrevistas semiestructuradas, realizadas a un representante de cada profesión con la que cuentan los dispositivos estudiados, correspondiendo a: una terapeuta ocupacional, una psicóloga, un asistente social, un psiquiatra y un técnico en enfermería de nivel superior. Todas las entrevistas semiestructuradas fueron grabadas en audio y transcritas, mientras que, para la observación participante y entrevistas etnográficas, se llevó a cabo un riguroso registro en el diario de campo, información que posteriormente fue transcrita. El material de campo producido por las distintas técnicas de investigación mencionadas fue sistematizado y sometido a un análisis de tipo temático. 

De este modo, las unidades de análisis quedaron constituidas por el total de trabajadores y profesionales que brindan atención en salud mental en los centros de salud participantes, los que correspondieron a cuatro técnicos en enfermería de nivel superior, una enfermera, un médico general y seis médicos psiquiatras, tres terapeutas ocupacionales, ocho psicólogos, tres asistentes sociales y cuatro secretarias.

Por otra parte, la escritura del presente texto la elaboramos de manera colaborativa haciendo uso de diversas voces; a veces, a partir de la narrativa de la primera persona singular, ya que el protagonismo en el trabajo de campo lo tuvo la investigadora principal y, en otros segmentos, usamos el plural de la primera persona o la tercera persona en singular, buscando dar cuenta de la reflexividad en el proceso de investigación y en el análisis y la escritura conjunta. De este modo, la reflexividad como estrategia de validez, fue una práctica habitual entre el equipo de investigadoras. Mediante la reflexividad, buscamos no caer en interpretaciones paternalistas frente a colegas o usuarios o, por el contrario, denostativas hacia el tipo de trabajo realizado por el personal de salud. Consideramos que dicho posicionamiento, permitió generar confianzas con los participantes, para profundizar en la comprensión del fenómeno de estudio. 

La validación desde la perspectiva de los participantes la realizamos a través de una jornada de devolución al equipo de salud, instancia en la que la investigadora principal, dio a conocer los resultados de la investigación, abriendo un diálogo y entregándoles el insumo producido, lo que fue bien acogido por el personal.

La investigación contó con los estándares éticos exigidos, tanto por el Comité de Ética de la Universidad de Concepción, como también por el Comité de Ética Científico del Servicio de Salud al que pertenecen los centros (orden No. 2164) al tratarse de una investigación con seres humanos en el ámbito médico. El estudio fue diseñado teniendo en cuenta los derechos de los participantes incluidos en La Declaración de Helsinki; por lo tanto, se contó con el consentimiento informado y voluntario de cada uno de los participantes, como también se aseguró la confidencialidad mediante el tratamiento anónimo de los sujetos y organismos involucrados. La protección de la información recabada estuvo a cargo de la investigadora principal, quien custodia los datos de manera anonimizada en un disco duro. Por otro lado, en congruencia a los estándares éticos solicitados por el Comité de Ética Científico, la investigación se enfocó solo en las perspectivas de los profesionales, lo que implicó tener un acceso a los usuarios solo en la relación establecida con el personal de la salud, y no de manera individual y autónoma.

El texto que presentamos surge de una investigación más amplia cuyo objetivo fue comprender cómo se presenta la estigmatización del personal de atención secundaria de salud mental hacia personas con diagnóstico de trastorno mental, en centros de salud mental de la Región del Biobío en Chile.

## Resultados

### Praxis en salud mental: la manifestación del estigma en la atención en salud mental secundaria

Con base en la investigación etnográfica realizada, en un trabajo anterior^21^ planteamos que la estigmatización hacia las personas con un diagnóstico de trastorno mental se desarrolla en su trayectoria de atención en salud, perpetuándose desde el funcionamiento y accionar cotidiano en los distintos niveles en que se organiza la atención en salud mental. Logramos pesquisar dinámicas de estigmatización vinculadas a los niveles macro, meso y microsociales que componen el sistema de atención, es decir, desde lineamientos estructurales del modelo de atención en salud, hasta prácticas cotidianas que manifiestan los profesionales que ejercen las labores en la atención secundaria. 

Proponemos que es en estos distintos niveles desde los que se gesta una concepción limitada sobre las personas que padecen un trastorno de salud mental; sin embargo, identificamos que la imbricación entre los distintos niveles del sistema de atención incide no solo en la estigmatización hacia los usuarios, sino también en la práctica profesional y en las concepciones laborales que tiene el personal de salud sobre su quehacer y su propia salud mental, incidiendo en la reproducción del estigma.

Esto se observa a nivel macrosistema en lo que hemos identificado como una tensión entre modelos de atención en salud mental; es decir que se produce una disonancia entre lo discursivo dado por el modelo comunitario y la praxis en la atención médica y clínica dada por el modelo biomédico. Aquella disonancia también repercute a nivel mesosistema, es decir, lo que identificamos en la práctica institucional cotidiana, mediante las formalidades y estructuras para brindar la atención; como también a nivel microsistema, en lo que hemos identificado como una institucionalización de la identidad de los usuarios y la repercusión que tiene para el personal de salud trabajar en salud mental. 

### Tensión de miradas entre el modelo comunitario y el enfoque biomédico de atención a los trastornos mentales

El personal de salud valora el modelo comunitario de atención en salud mental, señalando un mayor impacto de la intervención en salud mental desde dicho lineamiento. Así lo hicieron notar en las reflexiones ocurridas en el contexto de las reuniones clínicas, como fue el caso de una madre de un joven diagnosticado con esquizofrenia hace aproximadamente cuatro años. La madre indicó que el taller de fútbol en el que participa su hijo ha impulsado cambios positivos en este. Los profesionales, al analizar posteriormente el caso, opinan que los talleres que ofrece el centro diurno han “levantado” a muchos usuarios (Notas de campo 12 de junio, 2018). Sin embargo, independientemente del reconocimiento de cierta eficacia de las intervenciones comunitarias, los profesionales reconocen la preponderancia de lo que se entiende como parte del modelo de atención biomédico, es decir, centrado en el individuo y en lo farmacológico, tal como menciona una de las psicólogas en entrevista semiestructurada:

*Actualmente en lo que es sectores de salud mental -porque los COSAM los desaparecieron- por esta visión biomédica que impera estos últimos años, el psicólogo está en su oficina y ve un paciente tras otro y hace lo clásico que es de un psicólogo clínico, digamos: evaluaciones, terapia, diagnóstico, eso. Terapias más bien individuales, pocas grupales. Esto es una clínica donde la gente viene, pide su hora, ve a un profesional y se va; y ese profesional lo puede mandar donde otro y se va; y esos profesionales cada cierto tiempo tienen una reunión clínica donde hablan de este paciente y listo; eso no tiene el impacto que tiene un acercamiento comunitario.* (Entrevista con psicóloga, 7 de septiembre de 2018)

En la práctica clínica es posible apreciar la vigencia del modelo biomédico en salud sobre el comunitario, lo que se expresa principalmente en el abordaje de “los casos”, como son llamadas las personas que solicitan atención por los profesionales. Estos acostumbran a monitorear los avances de “los usuarios” y se alegran y sienten satisfacción cuando observan el progreso. No obstante, el análisis de estos avances se suele centrar en lo farmacológico, es decir, en cómo los medicamentos han ido generando cambios positivos en el usuario o en cómo lograr que lo hagan, llevando a enfocar el tratamiento en subir dosis, modificar el esquema o barajar la posibilidad de añadir fármacos que tienen muy buena reputación dentro de este contexto como, por ejemplo, la clozapina. Así, las mejorías son atribuidas mayormente a lo farmacológico, y los profesionales lo destacan durante la reunión clínica:

*Una vez que los usuarios abandonan la reunión clínica,* [los profesionales] *conversan sobre su evolución* [del usuario], *“está mucho mejor”, dice el psiquiatra y la psicóloga dice “ha cambiado su postura”, refiriendo a que el usuario mostraba menos rigidez corporal por la medicación*. […] *Revisamos el caso de un usuario que se encuentra internado en corta estadía, pero durante el día está en hospital de día. El psiquiatra le consulta sobre cómo está de apetito y sueño y pregunta por los “remedios”: “¿Quién te los da?”, a lo que el usuario responde que las personas de corta estadía. Cuando sale el usuario, se habla de su evolución, a lo que el psiquiatra comenta “qué ganas de darle clozapina, pero no tiene red de apoyo”, por lo que consulta a los colegas quién lo acompaña, le responden que su mamá, pero asistente social comenta “la madre es bien paranoide” y cuando consulta si es que ella recibe algún tipo de intervención, le dicen que no*. (Notas de campo, 3 de julio de 2018)

En el caso anterior, si bien el equipo médico pesquisa la necesidad de que la madre del usuario esté involucrada en el tratamiento, debido a la complejidad y las repercusiones en la salud del usuario que puede tener el uso de la clozapina, no existen intentos por abordar temáticas familiares o activar redes para que la madre pueda recibir intervención de tipo psicológico, pese a que ellos ya la tienen “diagnosticada” como “bien paranoide”. De esta manera. el equipo percibe que solo su “paciente” es quien ingresó formalmente, y así el trabajo con las redes cotidianas, es decir lo social, no alcanza a ser visto como una parte de la intervención, sino solo como un contexto que otorga pistas, de lo que farmacológicamente se puede hacer o no. En ciertas situaciones, los profesionales se reconocen como equipos “clínicos” a los que les falta la mirada y el abordaje psicosocial; sin embargo, cuando un caso requiere fundamentalmente este tipo de enfoque, perciben que no le corresponde al segundo nivel de atención, barajando la posibilidad de que sea atendido por el primer nivel. 

Dicha distinción entre casos clínicos y casos psicosociales implica que los primeros son los que deben ser atendidos por estos dispositivos de atención secundaria. Lo psicosocial, en cambio, no está integrado como aspecto esencial del sujeto y es percibido como un abordaje de menor complejidad. “*Es que nosotros somos muy clínicos*” es lo que responde la psicóloga, frente a la desazón del médico al señalar “*no creo que ya valga la pena que se siga atendiendo*”, refiriéndose al caso de una usuaria que no asume su diagnóstico psiquiátrico o “*sin conciencia de enfermedad*”, tal como lo catalogan los profesionales. A esto, la psicóloga añade que cree que es un caso de tipo psicosocial, por lo que la modalidad que tienen allí no se adecúa (Nota de campo, 9 de julio de 2018). 

Esta diferencia consignada entre “casos clínicos” y “casos psicosociales” impacta en la oferta de tratamiento que desde el propio dispositivo se hace, pues pareciera ser que la complejidad que amerita el ingreso al nivel secundario de salud mental, desestima las variables psicosociales, reduciendo las alternativas de respuesta del COSAM a barajar alternativas farmacológicas que remitan o disminuyan síntomas clínicos, o bien hospitalizaciones de los usuarios cuando esta sintomatología no responde a los fármacos. Así se comienza a ver cómo la complejidad psicosocial, tan presente en usuarios con trastornos de salud mental, se delega a la esfera del primer nivel de atención, introyectando una visión desde el propio dispositivo del segundo nivel, en la que se disocia la necesidad de intervención de las problemáticas comunitarias y psicosociales, que sustentan e incluso perpetúan la sintomatología. 

La tensión derivada de la coexistencia de ambos modelos permea el tratamiento, puesto que la fuerte presencia de la mirada biomédica lleva a que exista una naturalización de las explicaciones biológicas del comportamiento, lo cual contribuye a los prejuicios y discriminación^19^. Esto se vincula al carácter biologicista de la biomedicina, desde la cual el sujeto es abordado bajo una perspectiva reduccionista, al desprenderlo de su contexto social e histórico^13^^,^^14^, para sobrevalorar la explicación biológica de su padecimiento en desmedro a otras posibilidades que permitirían hacer frente al sufrimiento. 

Asimismo, esta tensión entre la visión comunitaria y la biomédica se ve facilitada por las diferencias que se dan entre los lineamientos institucionales que promueven un abordaje más comunitario y los recursos que existen para ello, como menciona una de las profesionales:

*Yo creo que las políticas macro en lo que es salud mental, no van por mal camino, sin duda, pero no se condicen con la realidad ¿entiendes? Entonces llegan acá manuales, destilados [risas], destilados del ministerio, y la verdad es que tú ves cosas que no se condicen ni con la población, ni con la realidad de la población, ni con los recursos y, por lo tanto, ni con la cantidad de funcionarios, porque los recursos, los funcionarios son parte de los recursos. Entonces hay muy pocos recursos, en realidad hay muy pocos recursos. Así que las ideas son buenas, pero la mayoría se quedan ahí no más*. (Entrevista con psicóloga, 7 de septiembre de 2018)

Los lineamientos institucionales comunitarios sobre el trabajo en salud mental son valorados, pero vistos como impracticables por su inadecuación a la realidad y, sobre todo, a los recursos profesionales. En este contexto, en la práctica de asistencia, lo biomédico puede ser priorizado pues requiere menos recursos humanos para su implementación ambulatoria, lo que deja a los equipos de salud en una constante escisión entre lo discursivo y su práctica profesional. En resumen, el enfoque biomédico se presenta principalmente a través de cómo son entendidas las intervenciones y cómo se mide su impacto. Dicha perspectiva hace que el éxito de la intervención sea evaluado a través de variables ligadas a lo conductual, que denoten disminución de los síntomas, dejando de lado aspectos sociales de la recuperación y enfatizando el poder que ha tenido el fármaco sobre la mejoría^32^. Este modelo condiciona cómo los profesionales se refieren a los usuarios y cómo actúan con ellos. En este sentido, se observa que las etiquetas diagnósticas son la guía indiscutible para el abordaje en salud, y las personas son homologadas a estas etiquetas^33^, generándose una visión reduccionista del usuario^34^^,^^35^. La etiqueta permite encarnar el rol de “paciente”, lo que también es generado desde las formas de organización y estructura institucional, como veremos a continuación.

### La praxis institucional del enfoque y modelo biomédico de atención

El modelo de atención utilizado tiñe la trayectoria de atención de los usuarios y prescribe formas de pensar y de tratarlos que pueden denotar estigma. El usuario, en primera instancia debe tomar el rol de “paciente” y reconocerse como “enfermo” de acuerdo con las apreciaciones clínicas que el equipo profesional define, junto con adherir al tratamiento que estos ofrezcan con base en el diagnóstico. 

Las dependencias de salud mental poseen un espacio exclusivo al interior del recinto hospitalario desde el año 2010. Estas fueron pensadas para ser usadas como centros comunitarios de salud mental (COSAM), destinados a la atención ambulatoria y también como hospitales diurnos. Las construcciones se conservan en muy buenas condiciones, poseen un estilo moderno, generando la sensación de brindar una atención médica actualizada y de calidad. En dichos lugares, existen salas para efectuar talleres y reuniones (Notas de campo, 12 junio de 2018). Si bien las edificaciones poseen un buen mantenimiento y se presentan como entornos agradables para la atención, en el espacio clínico las jerarquías rígidas que conforman la relación médico paciente se dejan entrever.

Para las reuniones clínicas se sitúa una mesa en el centro, en la cual el equipo médico se ubica en grupo formando una fila y, frente a ellos, la silla donde se ubica el usuario que ingresa a exponer sus afecciones y síntomas. Algunos profesionales ven cómo esta disposición física del espacio podría impactar negativamente en los usuarios, tal como reconoce uno de los psiquiatras en la entrevista:

…*lo que puede llegar a impactarles un poco más es eso, de repente llegar y encontrarse -si es que no lo han analizado a priori- con diez personas, un auditorio de personas en donde ellos están solos ahí, o a lo más con un acompañante, en que van a tener que exponer su caso, y su persona, en el fondo, a un equipo de diez. Pero yo te diría que eso, si no se les ha avisado, puede llegar a ser un impacto. Si se les ha avisado, es mucho menor*. (Entrevista con psiquiatra, 23 de octubre de 2018)

A su vez, el terapeuta ocupacional del COSAM cuando se le consultó en entrevista semiestructurada sobre su apreciación de las reuniones clínicas menciona: “*igual yo lo encuentro medio invasivo, entrar y que estén ahí, todos al frente y el usuario solo, contando sus cosas, además, como que igual podría ser diferente… una mesa redonda o no sé… algo más amigable*”. (Entrevista semiestructurada con terapeuta ocupacional, 7 de octubre de 2018)

Notamos que tanto las maneras de organizar el espacio, como la infraestructura del lugar, también van colaborando en la construcción del rol de “paciente” en salud mental, al ir generando diferenciaciones en los usos de los espacios, tal como sucede con la farmacia del centro de salud mental, la que es llamada en tono jocoso por diversos usuarios como “*la farmacia de los locos*”, conformado un punto de referencia para las personas que circulan por el lugar*.* Si bien el hecho de contar con una farmacia propia al interior del centro es visualizado como un recurso y como algo altamente positivo, por la comodidad que brinda a los usuarios su fácil acceso, también genera, a nivel social, una diferenciación entre el tipo de atención de salud general y el de salud mental.

Aquello nos lleva a considerar cómo las disposiciones materiales y espaciales van modificando las maneras en que el entorno percibe a los usuarios de salud mental. En este caso, se los separó de la farmacia general del recinto hospitalario y se los deja en un espacio social distinto, que se llega a reconocer de manera general como exclusivo para “*los locos*”. En este mismo sentido, podemos añadir que el espacio de la reunión clínica antes mencionada es otra instancia de interacción fundamental en la que desde el ingreso del “paciente” se remarcan las diferencias de jerarquía y de roles en la dinámica de la interacción clínica, ayudando a producir y acatar el etiquetamiento. 

Asimismo, las dinámicas de tensión en la praxis institucional no solo se ven afectadas por la infraestructura y equipamiento de los espacios, sino que también resultan relevantes las formas de administración y financiamiento de los centros, y así lo hace notar una de las profesionales entrevistadas:

…*en realidad hay muy pocos recursos* […] *porque, por ejemplo, acá debería haber muchas más residencias protegidas, muchas más, ¡y no hay residencias protegidas que sean del ministerio!, son residencias protegidas que son privadas, y como son privadas igual siempre esperan sacar una tajada. Entonces hay pacientes que han tenido que ser retirados porque no estaban bien atendidos, pagando además una buena, buen precio. Yo creo que debería haber más residencias protegidas; pero para eso también necesitas más personal del servicio público que pueda atender esas residencias protegidas, ¿me entiendes?, es por ponerte un ejemplo*. (Entrevista con psicóloga, 7 de septiembre de 2018)

En Chile las formas de administración y financiamiento de la salud mental se producen de manera mixta, concentrando la atención en los centros de salud de carácter público, pero recurriendo también a entidades privadas que reciben financiamientos públicos por medio de licitaciones. Estas formas burocráticas e institucionalizadas impactan en las experiencias y trayectorias de atención de los usuarios, como bien lo señala el personal de salud, condicionando las posibilidades de acompañamiento de los usuarios. Al respecto, Jenkins^36^, analizando la situación de EEUU, logra dar cuenta de las incidencias que provocan en las continuidades y discontinuidades de los tratamientos las modificaciones presupuestarias y administrativas de centros de salud mental, afectando en las posibilidades de recuperación. 

### La trayectoria de atención en salud mental como institucionalización de la identidad de los usuarios y afectaciones en el quehacer del personal de salud

En este nivel apuntamos directamente a los estereotipos, prejuicios y conductas estigmatizadoras que los profesionales ejercen hacia los usuarios y que contribuyen a una institucionalización de su identidad, la que comienza a asociarse casi en su totalidad, con el diagnóstico que se la ha asignado a la persona. Lo anterior, dificulta el proceso de intervención y de recuperación del sujeto usuario de salud mental. 

Asimismo, podemos observar que la aplicación de un tratamiento centrado, fundamentalmente, en un aspecto del usuario, como es la intervención farmacológica, favorece la “*cronificación de los pacientes*” en salud mental, entendida como la generación de una dependencia prolongada del usuario respecto a la atención en salud mental, derivada de intervenciones limitadas que no abordan la complejidad del contexto social del usuario^21^. La cronificación se relaciona con una percepción pesimista sobre las posibilidades de recuperación de los usuarios^37^. Esta visión influye en las alternativas de intervención que se ofrecen a la persona y merma su propia esperanza en salir adelante, lo que termina impactando en su recuperación. Las dificultades en la intervención y lo magro de los logros son consignadas por el personal como agotadoras, conduciéndolos a generar variadas estrategias de afrontamiento en pro de su bienestar.

### Los procesos de institucionalización de la identidad de los usuarios en la trayectoria clínica

La institucionalización de la identidad se da, principalmente, mediante el ejercicio del papel de “paciente” ligado al diagnóstico psiquiátrico, lo que se vincula, como hemos mencionado con anterioridad, a que el usuario debe asumir y demostrar su “conciencia de enfermedad”. 

En la reunión clínica, en general, los usuarios son emisores de sus vivencias, síntomas y afectaciones psicológico-psiquiátricas ante el equipo de salud, lo que los conduce a un rol mayoritariamente de receptores de las indicaciones y conclusiones profesionales; no obstante, en ocasiones, estos consultaban sobre los efectos de los medicamentos y los profesionales les respondían generalmente de una manera que desviaba la preocupación enunciada, o que devolvía al usuario la importancia de tomarse sus medicamentos, basándose en la ayuda que estos les brindarían. En la reunión clínica del 6 de agosto de 2018, el psiquiatra le responde a una usuaria que pregunta por dificultades para dormir, diciéndole que eso puede corregirse con un medicamento, a lo que ella indica que cree que quizás los medicamentos no la ayudan o le hacen mal, porque cree que ya son muchos, a lo que el psiquiatra le dice “*son solo dos tabletas, de salud mental al menos son esas*” cerrando la interacción, sin dar espacio a la usuaria para contrapreguntar o negociar el tratamiento. 

En este sentido, durante una entrevista al psiquiatra le consulté explícitamente sobre la actitud de los pacientes al ser receptores de prescripciones farmacológicas:

*Eh, a ver, yo creo que depende del paciente. Por eso, tú tienes que haber visto que hay pacientes que no, no tienen, digamos, mucha conciencia de lo que significa un tratamiento, las dosis. Entonces, por eso se implementa este sistema de los pastilleros, ¿no cierto?, en que lo trabaja la terapeuta ocupacional con los familiares y, a veces, con el mismo paciente. Ahora, nosotros tenemos una pequeña, entre comillas, desventaja en psiquiatría, que la polifarmacia es como la regla, es muy raro que un paciente esté con un solo medicamento; y eso no es porque queramos resolverlo todo con medicamentos, sino que porque la especialidad es así nomás. Entonces, de repente, claro, hay algunos pacientes que pueden llegar a sentir un cierto agobio, ¿no cierto?, respecto a tener como, no sé, tres o cuatro medicamentos que tomarse en el día y en distintas horas. Pero ese es un mal, llamémoslo así, o una característica propia de la especialidad; que yo diría que la polifarmacia es casi la regla. Y la monofarmacia es la excepción, o sea un paciente que está con un solo fármaco es la excepción*. (Entrevista con psiquiatra, 23 de octubre de 2018)

Esta minimización de la posición del usuario no solo se observa en la prescripción farmacológica, sino que permea toda la trayectoria de atención^38^, afectando las posibilidades de tratamiento y recuperación. Los usuarios enfrentan restricciones en su participación, lo que vulnera sus derechos en la atención de salud^39^^,^^40^. La percepción de los usuarios está ligada a prejuicios sobre sus capacidades; estos son vistos como inhábiles y, por tanto, necesitados de protección y cuidados lo que genera actitudes paternalistas y/o extremadamente compasivas^41^^,^^42^. Esta práctica no se condice con lo que se propone en teoría desde el modelo comunitario de atención en salud mental, que considera como un valor-principio la participación del usuario y su familia en el tratamiento, es decir, legitima el saber del otro desde una relación horizontal y dignificadora^43^. Por otra parte, el enfoque biomédico supedita la visión del usuario al equipo de salud, quienes son los que saben y, por tanto, acrecienta las jerarquías en un plano de diferencias de poder^44^, lo que no da cabida a la negociación de tratamiento. 

Es frecuente escuchar, en las instancias de reunión clínica, que los profesionales apelen a la necesidad de que el usuario cuente con “*conciencia de enfermedad*”, es decir, que la persona, una vez diagnosticada, debiera ajustar su conducta y concepción de sí misma, sobre la base de la etiqueta de salud mental asignada por el criterio profesional, que se sustenta principalmente en manuales estadísticos de enfermedades y problemas de salud como el CIE-10. Cuando la persona no posee “*conciencia de enfermedad*” el equipo profesional aboga para que surja, considerando que es un aspecto central para lograr éxito en la intervención. Ejemplo de ello es el intercambio en la reunión clínica, en el que la asistente social recuerda cómo se veía la usuaria antes y dice:

*Ahora se le ve más como paciente, antes venía por cosas puntuales nomás a preguntar acá, a lo que la psicóloga dice: “no había conciencia de enfermedad” y como equipo valoran el trabajo hecho por los profesionales del hospital de día sobre el tema, tanto con la usuaria como con la familia.* (Reunión clínica, 23 de julio de 2018).

*Otra usuaria en el contexto de la reunión clínica pregunta sobre la posibilidad de que la ansiedad que está sintiendo sea efecto de un medicamento y el psiquiatra le menciona: “la medicación te está ayudando, no perjudicando”. Acto seguido el médico le consulta sobre sus hijos y si se siente capacitada para cuidarlos, cerrando la entrevista y agradeciéndole que haya venido le indica: “hagamos caso”, lo que alude al hecho de la toma de fármacos.* (Notas de campo, 19 de junio de 2018)

En síntesis, puede apreciarse que, si bien la propuesta teórica apunta al abordaje comunitario, en la práctica suele mantenerse una fuerte tendencia a delegar el poder en el profesional, lo que promueve una mirada paternalista que menoscaba la autonomía y las capacidades del usuario, quedando este último supeditado a las indicaciones médicas, tal como puede apreciarse en las diversas situaciones de reunión clínica. 

El impacto del trabajo en salud mental en los profesionales y sus estrategias de afrontamiento

La complejidad de las situaciones de vida de los usuarios, que exceden las posibilidades de intervención desde lo farmacológico y requieren un abordaje psicosocial, sumado, por un lado, a la limitada oferta de intervenciones psicosociales y, por otro, a la constante tensión entre el modelo que se promueve discursivamente y aquel que se impone desde lo estructural y cotidiano, contribuye a generar en los profesionales una visión limitada acerca de sí mismos en su rol y su alcance en la intervención. Esto repercute en el ejercicio profesional, facilitando el agotamiento a nivel afectivo y cognitivo. El trabajo en salud mental va generando distintos sentimientos en los profesionales, entre los que predominan la pena y la rabia. En palabras de una profesional, lo que resulta más agotador para ella es:

*Que los casos que se ven son complejos, hay problemas de todo tipo y a mí la verdad es que me genera pena… a cualquiera le puede pasar tener una patología de salud mental, yo tengo hijos, no sé qué les pueda pasar más adelante, a veces doy gracias de que en mi familia está todo bien. Más que nada es eso, el trabajar con problemas de personas.* (Entrevista con asistente social, 10 de agosto de 2018)

La rabia, por otro lado, parece estar ligada a los usuarios que no siguen los requerimientos del equipo, lo que genera desgaste, en tanto la pena surge ante casos donde su situación vital es desfavorable. El psiquiatra, en una de las reuniones de equipo, analiza lo que les ocurre ante el caso de una usuaria (Ana) a la que se piensa nuevamente “*encuadrar*”, es decir, establecer, marcar o reforzar los límites y lo esperado como paciente de salud mental. Para ello, se la compara con otra usuaria (Berta), que estaba citada para ese mismo día y que igualmente faltó y que más o menos asiste con la misma intermitencia, pero que, pese a ello, no los ha llevado a replantear el trato y el “encuadre” que harán. En ese contexto el médico le pregunta al equipo:

*“¿Qué diferencia hay entre Berta y Ana?, que Berta nos genera pena y Ana nos genera rabia, tenemos que manejar eso, es parte del burnout de nuestro trabajo”. Frente a ello, los colegas comparten la apreciación y luego el psiquiatra suspira diciendo: “quedamos cansados con el caso… y solo con hablarlo”*. (Notas de campo, 25 de junio de 2018)

Existen instancias en que los casos conmueven a los profesionales y los afectan a nivel emocional, cuando por algún motivo la vivencia del usuario resuena con el profesional en un ámbito más personal. Como es el caso de una usuaria de 50 años, aproximadamente, que acude acompañada de su pareja. Posterior a la entrevista, el equipo comenta lo impactante del relato de la usuaria. Una psicóloga del equipo dice:

*...“a mí personalmente me afectó mucho sentir el sufrimiento de ella” y el médico comenta: “ella está sufriendo de verdad ah”, a lo que otra de las psicólogas participantes de la reunión añade: “ay a mí me da pena él, me acordé de un tío que está así, tan enamorado de su esposa y la acompaña y sufre porque ella no está bien”. Luego, una asistente social pregunta: “¿Qué hacemos?” Y el psiquiatra, que se integró a mitad de la entrevista, comenta que él cree que el diagnóstico efectivamente es una esquizofrenia.* (Notas de campo, 21 de agosto de 2018)

Los efectos que produce la pena en los profesionales también dependen de las estrategias de afrontamiento que cada uno posea y de cómo las implemente. Existen quienes la evaden y se enfocan en volver a lo práctico y definir alguna acción concreta respecto al usuario, mientras otros son movilizados por este sentimiento y se involucran más allá de lo esperado en su rol, como menciona una de las profesionales:

*A mí me da pena, se ve tanto problema, que a veces me afecta harto y eso mismo hace que trate de ayudar a veces más allá de lo que correspondería, por ejemplo, si veo un paciente con problemas y me voy dando cuenta que no tiene pega* [trabajo]*, tiene niños chicos, voy y le compro yo cosas, pero después le digo que lo gestioné con otros y se le mandaron a él ¿me entiendes? O a veces he ido a verlos a la casa así en mi vehículo nomás si ¿qué me cuesta? Y no es tanto por asistencialismo, es más que nada porque si yo puedo ayudar, ¿por qué no hacerlo?* (Entrevista con asistente social, 10 de agosto de 2018)

Este sentimiento puede llevar a los profesionales a tener actitudes positivas hacia los usuarios^45^^,^^46^, las que en ocasiones se traducen en excesiva compasión^42^. Por otra parte, el agotamiento cognitivo, alude al cansancio que genera la carga administrativa propia de trabajar en salud y, a su vez, al esfuerzo intelectual que implica el análisis clínico constante de los casos atendidos. Sumado a ello, el hecho de contar con una agenda copada constantemente por el gran número de usuarios que se deben atender produce que deban realizarse múltiples atenciones individuales por día, sin contar con mayor espacio para descomprimir y generar pausas entre cada una, lo cual es percibido como estar “*atomizados*” y se conceptualiza como algo que resulta desagradable.

Otro aspecto que también se incluye dentro de este tipo de agotamiento, son los efectos que producen actitudes de usuarios que sean categorizadas como de difícil manejo para el profesional como los casos de “*encuadre*” que mencionamos anteriormente. Resulta común que los profesionales mencionen “*me dejó agotado*”, tal como señala el psiquiatra, haciendo alusión a que un usuario estaba muy verborreico y eso dificultaba el poder controlar sus turnos de habla en la entrevista (Notas de campo, 19 de junio de 2018). De aquí desprendemos que lo que agota es aquello que no permite el fluir habitual de la dinámica de entrevista y que demanda que el profesional deba modificar su actuar, ajustándose a la situación, lo que implica un mayor esfuerzo. Esto puede impactar en que se extiendan las jornadas de reunión pues las situaciones son complejas de abordar.

El agotamiento también es producido por las distintas destrezas que deben desplegarse al trabajar en salud mental. Esta es un área de desempeño en la que se debe poner mucha atención sobre qué se hace y cómo se hace, por ende, requiere contar con competencias tanto técnicas como socioemocionales. El trabajo se percibe como difícil por lo demandante, y se necesitan capacidades que exceden lo habitual. Sumado a ello, en este trabajo, se requiere ser integral, en el sentido de que haya un equilibrio entre lo técnico y lo personal. Es decir, requiere prestar atención tanto al entorno como a sí mismo, lo cual en ocasiones vuelve desgastante la labor y está claramente plasmado en las palabras de uno de los profesionales durante la entrevista semiestructurada:

*Aparte de tener que estar pendiente de la cosa técnica, de lo que es formular la hipótesis diagnóstica, hacer el planteamiento del tratamiento, hay que también estar muy atento a uno mismo, ¿no cierto?, a lo que a uno le está pasando… porque los pacientes generan emociones, generan, no cierto, lo que se conoce como “contratransferencia”. Entonces es un trabajo doble, ¡un trabajo doble que también se hace con los otros pacientes! También uno tiene que estar pasando: “A ver, bueno, este es esquizofrénico, ¿estoy metiéndome realmente en su caso clínico?, ¿estoy logrando llegar a lo…?”, si es que eso no ocurre. Pero es mucho menos dificultoso que en este otro caso, en los otros casos de trastornos de personalidad, por ejemplo, en que tú tienes que echar mano a todas tus herramientas terapéuticas para mantener la neutralidad que es sumamente necesaria para lograr el éxito de estos casos. Ahora, dependiendo de la experiencia, de la edad, del tiempo que uno lleve, esto se tiende a hacer de manera más automática, ¿ya? Pero cuando uno, de repente, está entrampado o sientes que como que algo está pasando que no está funcionando, tienes que detenerte un poquito y empezar a ver: “bueno, a ver, ¿qué me está pasando?, ¿cuál es la contratransferencia?* (Entrevista con psiquiatra, 23 de agosto de 2018)

El agotamiento producido por trabajar en salud mental, sobre todo lo emocional, es reconocido por los profesionales como un “*gaje del oficio*”, es decir, como parte de la responsabilidad que les toca asumir como trabajadores de la salud. El agotamiento emocional sería un efecto inevitable, al tener que espectar y/o conectar continuamente con sintomatologías, comportamientos y/o condiciones psicosociales adversas, que denotan el sufrimiento de las personas que atienden. 

Por ello, se han implementado ciertas estrategias que permitan la descompresión en los trabajadores, generando instancias que denominan de “autocuidado”, las cuales tienen una frecuencia mensual, en la que destinan media jornada a hacer algo juntos de carácter extralaboral como, por ejemplo, salir a comer a un espacio externo distinto. Esta instancia es financiada de forma mixta, con aportes desde la institución y desde los trabajadores, buscando crear interacciones desde la distención y poder brindar así espacios de relajo compartido. 

Por otra parte, frente al agotamiento descrito, otro de los aspectos que los profesionales reconocen y valoran como un factor protector son los espacios de interacción existentes entre colegas dentro de la praxis laboral, como los análisis conjuntos que se llevan a cabo en las reuniones clínicas. Dicha instancia laboral, se vive también como un espacio para el llamado “autocuidado”, destacando que allí pueden hablar de lo que les sucede con determinado usuario y, en ocasiones, también emplear sentido del humor entre colegas, como forma de limitar la carga afectiva que acarrea su trabajo. 

No obstante, se observa que en algunos profesionales este agotamiento emocional, al ser cotidiano y continuo, como un mecanismo de defensa, desemboca en que se desconecten de la emocionalidad y sufrimiento que presentan las personas atendidas. Lo anterior lleva, en algunos casos, a una estandarización y automatización de los procesos de atención e intervención, ya que se tiende a percibir como homologable, la conducta, el comportamiento y los síntomas de los distintos usuarios, limitando de este modo, que se pueda hacer un ejercicio de comprensión fenomenológica de la experiencia de cada persona que busca atención. 

Asimismo, consideramos que esto puede desensibilizar al profesional, quien tenderá a enfocarse más en el síntoma y el diagnóstico que en la experiencia individual de la persona. Así se incrementa el riesgo de deshumanizar los procesos de atención y de perpetuar el estigma, al reforzar estereotipos sobre cómo puede y debe ser un usuario de salud mental. 

## Discusión

Los hallazgos presentados en esta investigación permiten reflexionar sobre las tensiones y contradicciones que emergen entre el enfoque biomédico y las perspectivas en salud mental comunitaria. Particularmente, se ponen en evidencia las dificultades y las convergencias entre un discurso institucional que no se condice necesariamente con la práctica de atención en salud mental^20^^,^^21^. Observamos que la implementación del modelo comunitario de atención en salud mental, si bien forma parte de una adecuación de los servicios de salud mental a las desigualdades y diversidad de la realidad sociocultural^47^, aún no logra consolidarse plenamente^48^. 

Esto se sostiene en la oposición entre el modelo biomédico de atención y el modelo comunitario. Esta tensión permea no solo el tratamiento de las personas que padecen un trastorno en salud mental, sino que también afecta el ejercicio laboral, médico y de atención que realiza el personal de salud mental^20^^,^^48^. En el plano declarativo, la atención se guía por un modelo comunitario de salud mental, mientras que, en la práctica, sigue primando la perspectiva biomédica que conforma el modelo médico hegemónico^13^^,^^14^ que, en el ámbito de la salud mental, basa su modelo explicativo en la enfermedad y los desequilibrios neuroquímicos, por sobre los determinantes sociales que pueden incidir en los padecimientos^49^, naturalizando las causas biológicas del comportamiento y contribuyendo a la reproducción de prejuicios hacia las personas diagnosticadas en salud mental^19^. 

La primacía de lo biomédico sobre lo comunitario^50^ repercute en la tensión identificable entre el abordaje clínico *versus* el psicosocial, siendo este último asociado a lo comunitario. Aunque valorado por el personal de salud mental, dicho enfoque es percibido como difícilmente aplicable frente a la realidad social y económica de la población atendida, así como por las limitaciones de recursos económicos y profesionales con que cuentan los servicios de atención. Esto puede traducirse en que el contexto social en el que vive el usuario aporta antecedentes relevantes para determinar el tipo de tratamiento farmacológico; sin embargo, la dimensión psicosocial no es integrada como un aspecto esencial del sujeto, sino que se la considera como un elemento secundario en el ejercicio terapéutico^49^. 

Asimismo, las dificultades o contradicciones mencionadas forman parte de desigualdades estructurales que implican el sometimiento del personal de salud mental a una sobrecarga laboral^47^. Tal como emerge en el estudio, el trabajo en salud mental implica un desgaste persistente en quienes lo ejercen, fenómeno ampliamente señalado en la literatura^51^. El denominado síndrome de burnout, entendido como una experiencia de agotamiento emocional y desgaste profesional, aparece con particular fuerza en el personal de salud mental^52^. Sin embargo, más que un rasgo individual, este agotamiento se configura en relación con las condiciones mismas de la práctica: atender a personas cuyas problemáticas son complejas y de difícil resolución, y los avances se perciben como lentos o casi imperceptibles, lo que instala en los equipos la sensación de límite e impotencia^51^^,^^53^.

En este marco, los usuarios que se resisten a las lógicas institucionales -aquellos que no se ajustan a los encuadres o a las prescripciones biomédicas- tensionan aún más la labor cotidiana. Lejos de ser meros “pacientes difíciles”, encarnan el recordatorio de que las normas del sistema no siempre logran contener la diversidad de trayectorias vitales, lo que a la vez incrementa el esfuerzo requerido por los equipos y expone a estos usuarios a mayores riesgos de ser estigmatizados^54^.

El agotamiento del personal de salud mental también se nutre del contacto constante con el sufrimiento humano en un contexto de demandas técnicas y personales elevadas. Aunque son reconocidos como especialistas, muchos perciben no disponer de las herramientas necesarias para sostener este trabajo, lo que refleja la tensión entre las expectativas sociales depositadas en su rol y las posibilidades reales que ofrece el sistema. Esa tensión se agrava en escenarios como el chileno, donde la salud mental recibe históricamente escasos recursos: en 2021, apenas un 1,7 % del presupuesto de salud fue destinado a este ámbito^55^. Esta precariedad estructural se traduce en sobrecarga, agendas copadas y demandas imposibles de abarcar, reforzando un ciclo de desgaste y malestar que ha sido descrito también en otros contextos^56^^,^^57^.

Es importante subrayar que el personal de salud mental no permanece pasivo frente al agotamiento, sino que despliega distintas estrategias para afrontarlo. En este estudio, una de las más relevantes fue el recurso al apoyo social en el entorno laboral, entendido como una forma de enfrentar el estrés que ofrece ventajas significativas a quienes la utilizan^58^^,^^59^. Espacios institucionalizados como las instancias de “autocuidado” o las reuniones clínicas adquieren aquí un valor particular: más allá de su dimensión técnica, se constituyen en lugares donde los profesionales pueden socializar con sus pares, compartir experiencias y poner en común las emociones que suscita el trabajo cotidiano. Estos momentos, al habilitar la circulación de la palabra y la empatía entre colegas, cumplen una función de contención y alivio frente a las tensiones del día a día^60^.

Otra estrategia que emerge con fuerza es la insensibilización frente a los problemas de los usuarios^61^^,^^62^. Si bien permite a los profesionales crear una cierta distancia emocional que amortigua el impacto del sufrimiento ajeno, sus efectos se trasladan directamente al vínculo con quienes reciben la atención. Esta forma de afrontamiento puede erosionar la relación terapéutica, repercutiendo en la adherencia al tratamiento, la satisfacción con la atención y, en última instancia, en los propios procesos de recuperación^63^. El círculo se cierra cuando la frustración del personal de salud mental ante la percepción de estancamiento o cronificación de algunos usuarios alimenta nuevas formas de estigmatización: los usuarios son vistos como responsables de su falta de avance, lo que refuerza actitudes negativas hacia ellos y tensiona aún más el horizonte de la recuperación, lo que habría que seguir investigando.

## Conclusiones

El estigma hacia las personas con diagnóstico psiquiátrico por parte del personal de salud se ancla en la trayectoria de atención en salud mental. Esta trayectoria se ve cruzada por aspectos macrosociales ligados a la tensión entre dos modelos de atención: el modelo comunitario de atención en salud mental y el modelo biomédico. El primero es impulsado por las políticas de salud mental del país^7^, en tanto el segundo está incrustado en las prácticas habituales de trabajo. Los determinantes sociales de la salud mental, en la praxis cotidiana, son escasamente abordados, quedando lo psicosocial supeditado a lo biomédico. Este modelo comprende y aborda el sufrimiento de forma tal que el saber técnico asociado al diagnóstico y la prescripción de medicamentos es lo central, lo que tiene como consecuencia, por una parte, que saberes de otros profesionales sean subvalorados y, por la otra, que en la atención se resta participación a las personas diagnosticadas en su tratamiento. Restar protagonismo al usuario es una forma de estigmatización, pues denota una visión que menoscaba sus capacidades y potencialidades, lo que puede afectar su recuperación, contribuyendo a su cronificación. 

Por otra parte, el modelo biomédico, al enfatizar el diagnóstico y el tratamiento farmacológico, prioriza la necesidad de que el usuario tenga “conciencia de enfermedad”, entendida como la aceptación de su identidad de “paciente” y adopte las prescripciones médicas sin cuestionamiento. De modo que, las presiones ejercidas por el equipo para seguir el tratamiento pueden ser consideradas como coerción, una de las formas en que se ejerce el estigma^64^. 

Las tensiones entre estos modelos también se expresan en la forma en que el sistema de salud organiza y administra los recursos. Se predica por la aplicación de un modelo comunitario de atención en salud mental pero, de acuerdo a los profesionales, se asignan recursos insuficientes para su implementación, mientras que al personal de salud mental se le exige de acuerdo a parámetros centrados en la consulta individual. En el ejercicio de su cometido, el personal de salud se escinde entre lo solicitado y sus posibilidades reales de cumplimiento. Esta situación contribuye a generar en los profesionales una visión limitada acerca de sí mismos, en su rol y su alcance en la intervención; lo que sumado a las dificultades que conlleva trabajar con el sufrimiento humano, en un rol donde las demandas técnicas y personales que requiere su ejercicio son altas, parece inevitable el desgaste profesional. Este, a su vez, impacta en la relación con los usuarios, lo que facilita la estigmatización^65^. No obstante, el personal de salud mental ha desarrollado estrategias de afrontamiento que le facilitan sobrellevar estos desafíos, es decir, tiene un papel activo. Hay estrategias más personales, como la evitación, y otras más colectivas, basadas en lo que los equipos denominan como “autocuidado”. Este cuidado es una estrategia centrada en poder liberarse de las tensiones cotidianas del trabajo, afianzando las relaciones de confianza entre el personal de salud a través de una actividad generalmente de recreación. Por su parte, las reuniones clínicas no solo tienen una connotación técnica, sino que representan la posibilidad de contar con apoyo social en el entorno laboral, y desde esa perspectiva son parte de estrategias de cuidado entre el personal de salud. En un ambiente laboral donde se trabaja en equipo, es decir, de manera colaborativa, el cuidado no puede ser una tarea individual, sino que debe -y en la práctica lo es- una labor colectiva. Cuidarnos implica confianza, reciprocidad y apoyo mutuo. De esta manera, se pueden enfrentar mejor los retos que supone un trabajo agotador. 

En esta investigación, el estigma, más que asociado a estereotipos o actitudes individuales, aparece anclado en aspectos macro, meso y microsociales, dados por el modelo de atención y la estructura del sistema de salud, lo que impacta en las prácticas cotidianas de los profesionales, particularmente en su conceptualización de lo que les sucede a las personas con diagnóstico en salud mental y cómo abordarlo. Por tanto, si queremos disminuir la estigmatización, es importante que miremos el sistema donde se genera y pensemos en intervenciones que impliquen incorporar modificaciones en su interior. Mientras sigamos abogando por una mejora en las actitudes del personal de salud mental hacia sus usuarios y olvidemos el entramado social y político que da sentido a estas actitudes y conductas estigmatizantes, los cambios serán acotados. Además, es fundamental considerar las repercusiones que tiene para el personal de salud trabajar en salud mental, es decir, los desafíos que presenta esta labor deben ser considerados, no solo para prevenir el agotamiento de los profesionales, sino también para reducir las repercusiones en el trato interpersonal con los usuarios, propio del desgaste laboral. 
