# Supplementary material for: La praxis de la atención en salud mental y la manifestación del estigma: un estudio etnográfico en centros de salud mental de la Región del Biobío, Chile
Source: Salud Colect. 2025 Oct 8;21:e5631. [Article in Spanish] doi: 10.18294/sc.2025.5631 (PMC13356486; doi:10.18294/sc.2025.5631)
Supplement: Translation

# Translation: The praxis of mental health care and the manifestation of stigma: an ethnographic study in mental health centers in the Biobío Region, Chile

**Authors:** Romina Jara-Ogeda, Pamela Grandón Fernández, Daniela Leyton Legües

**Affiliations:** 1 Magíster en Psicología. Jefe técnica, programa Familias de Acogida Especializada, FAE Ayün, Corporación de Apoyo a la Niñez y Juventud en Riesgo Social Llequén, Los Ángeles, Chile. psromina.jarao@gmail.com; 2 Doctora en Psicología. Profesora titular, Departamento de Psicología, Universidad de Concepción, Concepción, Chile. pgrandon@udec.cl; 3 Autora de correspondencia: Doctora en Ciencias Humanas. Profesora asociada, Facultad de Ciencias Sociales, Universidad de Concepción, Concepción, Chile. danielaleyton@udec.cl

**Keywords:** Mental Health, Mental Disorder, Stigma Social, Health Care System, Ethnography, Chile

## Abstract

In this article, we inquire into how mental health praxis reproduces stigma toward users of mental health centers in the Biobío Region of Chile, focusing on the relational space of interactions where stigma is expressed and sustained. Based on a qualitative research design with an ethnographic approach, from June to September 2018 we conducted participant observation in both formal and informal contexts of health services, as well as ethnographic and semi-structured interviews. We identified a persistent tension between the community mental health model promoted by public health policies and biomedical approaches to the care of users with psychiatric diagnoses. These tensions shape the daily practice of health personnel and, consequently, the type of interventions that users receive in their interactions and participation within health services. They manifest in at least two dimensions: first, in the predominance of the biomedical model over other medical practices, which impacts and constrains the exercise of professional roles; and second, in the working conditions faced by mental health professionals, which lead them to experience burnout. We argue that both dimensions, in their interrelation, influence trajectories of mental health care and contribute to the reproduction of stigma toward people living with mental health conditions.

## Introduction

People diagnosed with psychiatric disorders are frequently subjected to stigma, which has multiple and negative consequences for those affected.^1^ Stigma refers to the process of labeling, status loss, and discrimination against individuals who exhibit a socially devalued characteristic.^2^ According to Goffman,^3^ the social construction processes are essential to understanding stigma. It is shaped through negative attributes that devalue the individual, reducing them to those traits and relegating other defining characteristics to the background. This reduction allows the stigma to become perceived as the person’s core identity trait. This process of internalizing stigma, along with the behavior that aligns with the role of the patient, has been described by Goffman as the “moral career” of the mentally ill. This concept reveals how, beyond clinical treatment, health care personnel contribute - consciously or unconsciously - to the social construction of stigma and to the imposition of a restricted role on people with psychiatric diagnoses.

When individuals are primarily defined by their diagnosis, and representations associated with chronicity, incapacity, or dangerousness are reinforced within daily health care practices, mental health professionals in secondary care settings often reproduce stigma toward people with psychiatric diagnoses.^4^ In this way, the conceptions held by mental health teams about their users are materialized: how they interpret what is happening to them and how they address their situations, the care model they hold and apply in their practice.^5^

In Chile, mental health policy is based on the community mental health care model, understood as a set of principles and practices aimed at promoting the mental health of groups and communities, primarily through the organization and structure of health services.^6^ In the country’s public system, secondary-level services provide care for individuals diagnosed with more complex mental disorders through Outpatient Psychiatry Units, Community Mental Health Centers (COSAMs), and Day Hospitals.^7^ These health services are part of the public sector, which serves approximately 80% of the country’s population through the National Health Fund (FONASA), a public health insurance system.^8^ FONASA covers a large proportion of older adults and vulnerable populations. Its coverage is higher among women (81.2%) than men (76.6%), and it exceeds 90% in the two lowest income quintiles, while in the highest quintile, coverage drops to 37.9%.^9^

Following recommendations from the World Health Organization (WHO), these services should be located in the communities where users live, integrated into general health care services, and include multidisciplinary teams. From this perspective, users are viewed as integral and autonomous individuals, whose conditions are understood not only as biological but also psychosocial in origin.^10^ In this view, people diagnosed with mental disorders live in socially vulnerable contexts and present a range of cognitive, emotional, and relational capacities that make it difficult for them to cope with challenges.^11^ This approach is expected to reduce intolerance and prejudice and increase empathy and the willingness to support individuals with mental health diagnoses.^12^

Nevertheless, in practice, the biomedical model of care continues to predominate. According to Menéndez,^13^^,^^14^ biomedicine has been established as the hegemonic medical model (HMM) over other ways of understanding health, illness, and care processes, as a result of specific historical, socioeconomic, and political developments in Western societies. The HMM is characterized, among other aspects, by a hierarchical doctor-patient relationship, by the individualization and reduction of the subject through the detachment from their sociohistorical context, and by an exacerbated biologicism.^13^^,^^14^

In the field of mental health, the HMM approaches so-called mental disorders from a biomedical logic in which these are explained as brain dysfunctions -that is, as illnesses based on genetic and neurobiological abnormalities. As such, they are considered to be of the same nature as physical illnesses. Consequently, their treatment is based on proper diagnosis and pharmacological prescription, which involves identifying signs and symptoms of the pathology.^15^ In this way, the individual’s suffering is individualized and stripped of its sociocultural context.

Within this hierarchical approach, the physician - specifically the psychiatrist - holds the highest status, followed by the psychologist.^16^ Both “psy” professionals are socially legitimized to make psychological or psychiatric diagnoses, while medical prescriptions are the exclusive responsibility of the psychiatrist. In this model, the person ends up being monitored based on the presence or absence of symptoms,^17^ which are matched against those listed in the DSM-5 diagnostic manual. The emphasis on diagnosis and medication is so significant that even other professionals, despite having alternative perspectives on users, end up prioritizing these aspects in their practice.^16^

Among health personnel, it has been found that this model increases the desire for social distance, reduces empathy, and interferes with the therapeutic relationship with mental health users,^18^ which can lead to negative attitudes and the dehumanization of these individuals.^19^^,^^11^

Therefore, mental health teams make use of diverse care models, including the community mental health care model and the HMM, which often contradict each other in how they conceptualize users and approach their experiences. Mental health professionals are expected to adopt a comprehensive understanding of users in line with the community model; however, in practice, they are compelled to operate within the logic of the HMM. This contradiction generates tension and ultimately influences their work experience.^20^^,^^21^ We therefore argue that it is important to consider the impact of mental health work on professionals themselves, and to pay attention to their needs in order to prevent harm to their own mental health.^22^

In Chile, research on stigma toward mental disorders among health professionals remains limited. Most studies have focused on primary care professionals, who have been found to express rejection and distrust, minimize users’ physical symptoms, and perceive them as dangerous.^23^^,^^24^^,^^25^ The few studies involving specialized mental health personnel show that they also stigmatize users, but do so through interactions that undermine and restrict users’ rights and opportunities to participate in treatment, thereby hindering recovery.^26^^,^^27^ These findings are still incipient, which leads us to conclude that further research is needed to understand how stigma manifests within this group of workers -especially considering that stigma reduction is part of the National Mental Health Plan.^7^

### Ethnographic entry into mental health centers in the Biobío Region

We grounded the implementation of ethnography for this research in the need to explore the manifestation of social stigma from the perspective of professionals in their everyday work context, which allowed us to describe situations based on the observation of the actors’ own practices.^28^ At the same time, we sought to investigate the ideas, beliefs, meanings, knowledge, and practices shared by a particular group,^29^ in this case, those deployed in the daily work context of health professionals employed in secondary-level public mental health care centers in the Biobío Region. From an ethnographic perspective, we developed a contextualized description based on participant observation,^28^ focusing on the social interactions among professionals. The ethnographic data emerge from our team’s analysis and interpretation of the information generated through observations, interviews, ethnographic conversations, and semi-structured interviews.^29^

The ethnographic fieldwork was conducted in two Community Mental Health Care Centers (COSAM) and the one day hospital located in the Biobío Region, in south-central Chile. These sites were selected because they serve as reference centers for the treatment of individuals diagnosed with mental disorders, and all three agreed to participate in the study. Fieldwork was carried out over a three-month period, from June 11 to September 11, 2018, with the weekly time allocated to participant observation distributed evenly across the three centers.

As a research team, we shared an understanding of knowledge production grounded in constructivist and critical paradigms, which hold that realities are locally and contextually constructed, while recognizing the structural conditions that shape them. Interactions between researchers and participants took place at different levels of collaboration, enabling us to approach the phenomenon under study as a co-created process. At the same time, consistent with critical perspectives, we acknowledge that findings are mediated by values -that is, our own positions and histories as subjects inevitably shaped the research process.^30^ In this regard, it is worth noting that access to the field was facilitated by the main author’s professional training in psychology, as well as her prior internship in a nearby health center, which encouraged professionals to participate voluntarily and allowed for the observation of their everyday interactions.

The research techniques employed to produce the ethnography included three months of participant observation, ethnographic interviews in informal contexts of interaction with professionals, and semi-structured interviews. Participant observation took place in spaces of interaction among professionals, and between professionals and individuals seeking care. Participation occurred in formal settings, such as clinical team meetings, intake meetings in the day hospital, group activities, and home visits, as well as in informal everyday situations, such as professionals’ lunch breaks. Observations were complemented by ethnographic interviews, understood as conversations between the researcher and participants that emerged casually in the context of observation - without being previously defined, yet guided by the research objectives.^31^ These conversations were carried out throughout the fieldwork in order to open and identify emerging topics and to deepen issues that arose during observations.

The semi-structured interviews were conducted in order to deepen the emerging themes identified during ethnographic fieldwork, such as: experiences of working in mental health; perceptions regarding clinical team meetings; personal assessments of the functioning of the mental health pharmacy and of drug prescription as a health care practice; conceptualizations and approaches to “complex cases”; and, finally, descriptions and perceptions of workshops offered and home visit practices. Five semi-structured interviews were carried out, one with a representative of each profession working in the studied facilities: an occupational therapist, a psychologist, a social worker, a psychiatrist, and a higher-level nursing technician. All semi-structured interviews were audio-recorded and transcribed, while participant observation and ethnographic interviews were documented in detail in a fieldwork diary, which was subsequently transcribed. The field material generated through these different research techniques was systematized and analyzed using thematic analysis.

The units of analysis were therefore composed of the total number of workers and professionals providing mental health care in the participating centers: four higher-level nursing technicians, one nurse, one general practitioner and six psychiatrists, three occupational therapists, eight psychologists, three social workers, and four administrative secretaries.

The writing of this article was carried out collaboratively, incorporating multiple voices. At times, the narrative is expressed in the first person singular, reflecting the central role of the main researcher in the fieldwork; in other instances, we employ the first person plural or third person singular, as a way to account for reflexivity in the processes of research, analysis, and collective writing. Reflexivity, as a strategy of validity, was a constant practice within the research team. Through reflexivity, we sought to avoid paternalistic interpretations of colleagues or service users, as well as disparaging views of the work carried out by health care staff. We consider that this positioning allowed us to build trust with participants, thereby deepening the understanding of the phenomenon under study.

Validation from the participants’ perspective was pursued through a feedback session with the health care team, during which the main researcher presented the study’s findings, opening a dialogue and sharing the research outputs. This initiative was well received by the staff.

The study complied with the ethical standards required both by the Ethics Committee of the University of Concepción and by the Scientific Ethics Committee of the Health Service to which the centers belong (Order No. 2164), given that it involved research with human subjects in the medical field. The study was designed in accordance with the rights of participants outlined in the Declaration of Helsinki. Accordingly, informed and voluntary consent was obtained from all participants, and confidentiality was ensured by anonymizing the data of individuals and institutions involved. The protection of the collected information was the responsibility of the principal researcher, who safeguarded the anonymized data on a secure hard drive.

Furthermore, in line with the ethical standards established by the Scientific Ethics Committee, the research focused exclusively on the perspectives of health professionals. This meant that access to service users occurred only through their interactions with health care staff, and not individually or autonomously.

The present text derives from a broader research project aimed at understanding how stigmatization is expressed by secondary-level mental health care staff toward individuals diagnosed with mental disorders in mental health centers in the Biobío Region of Chile.

## Results

### Praxis in mental health: The manifestation of stigma in secondary mental health care

Based on ethnographic research conducted, in a previous study^21^ we argued that stigmatization toward individuals diagnosed with a mental disorder develops throughout their trajectory of care, and is perpetuated through the daily functioning and practices at the different levels in which mental health care is organized. We identified stigmatizing dynamics linked to the macro, meso, and micro-social levels that compose the health care system -ranging from the structural guidelines of the model of mental health care to the everyday practices enacted by professionals working in secondary care.

We propose that it is across these different levels that a restricted conception of people living with a mental disorder is produced. However, we also identified that the interplay between the various levels of the health care system affects not only the stigmatization of service users but also the professional practice itself and the ways in which health workers conceive of their own roles and their own mental health, thus contributing to the reproduction of stigma.

At the macrosystem level, this is evident in what we identified as a tension between models of mental health care: a dissonance arises between the discourse of the community model and the praxis of medical and clinical care framed within the biomedical model. This dissonance also reverberates at the mesosystem level, that is, within the everyday institutional practices and structures through which care is delivered; as well as at the microsystem level, where we observed an institutionalization of users’ identities and the repercussions this has for health professionals working in mental health.

### Tension between the community model and the biomedical approach to mental disorders

Health professionals value the community model of mental health care, highlighting the greater impact of interventions guided by this approach. This was made evident in the reflections that took place during clinical meetings. For example, the mother of a young man diagnosed with schizophrenia about four years ago noted that the soccer workshop in which her son participates had promoted positive changes in him. When discussing the case afterwards, professionals commented that the workshops offered at the day center had “lifted up” many users (Fieldnotes, June 12, 2018).

Nonetheless, despite acknowledging the effectiveness of community interventions, professionals also emphasized the predominance of what is understood as part of the biomedical model of care - that is, one centered on the individual and on pharmacological treatment - as one psychologist pointed out in a semi-structured interview:

*Currently, in what are mental health sectors -because the COSAMs were eliminated - due to this biomedical vision that has prevailed in recent years, the psychologist is in their office and sees one patient after another and does the classic thing of a clinical psychologist, let’s say: evaluations, therapy, diagnosis, that. Mostly individual therapies, few group ones. This is a clinic where people come, book an appointment, see a professional and leave; and that professional can send them to another one and they leave; and those professionals, every so often, have a clinical meeting where they talk about this patient and that’s it; that does not have the impact that a community approach has.* (Interview with psychologist, September 7, 2018)

In clinical practice, the predominance of the biomedical model of health care over the community model becomes evident, particularly in the approach to “cases,” as professionals refer to the individuals seeking care. Professionals usually monitor the progress of “users” and express joy and satisfaction when improvements are observed. However, the analysis of such progress is often centered on pharmacological aspects - that is, on how medications have generated positive changes in the user, or on strategies to ensure they do so. This frequently leads to treatment decisions focused on increasing dosages, modifying treatment regimens, or considering the addition of drugs that enjoy strong reputations in this context, such as clozapine. In this way, improvements are attributed primarily to pharmacological treatment, and professionals underscore this during clinical meetings:

*Once the users leave the clinical meeting,* [the professionals] *talk about their progress* [the user’s], *“he is much better,” says the psychiatrist, and the psychologist says “his posture has changed,” referring to the fact that the user showed less body rigidity due to the medication*. […] *We review the case of a user who is hospitalized in short stay, but during the day attends the day hospital. The psychiatrist asks him about his appetite and sleep and asks about the “medications”: “Who gives them to you?” to which the user replies that the short-stay staff do. When the user leaves, they talk about his progress, to which the psychiatrist comments, “I’d really like to give him clozapine, but he has no support network,” so he asks his colleagues who accompanies him. They answer that his mother does, but the social worker comments, “the mother is quite paranoid,” and when he asks if she receives any kind of intervention, they say no*. (Fieldnotes, July 3, 2018)

In the previous case, although the medical team recognized the need for the user’s mother to be involved in the treatment - given the complexity and health implications associated with the use of clozapine - there were no attempts to address family-related issues or to activate support networks so that the mother could receive psychological intervention, even though they had already “diagnosed” her as “quite paranoid.” In this way, the team perceived that only their “patient” had formally entered the system, and thus everyday social networks were not regarded as part of the intervention but rather as a contextual source of clues about what could or could not be done pharmacologically.

In certain situations, professionals recognized themselves as “clinical” teams lacking a psychosocial perspective and approach. However, when a case was deemed to require this type of focus, they tended to see it as outside the scope of secondary care, suggesting instead that it should be managed at the primary care level.

This distinction between “clinical cases” and “psychosocial cases” implied that only the former should be attended to within these secondary-level services. The psychosocial dimension, by contrast, was not integrated as an essential aspect of the individual and was perceived as an approach of lesser complexity.

“*It’s just that we are very clinical*,” responded a psychologist when the physician, disheartened, stated: “*I don’t think it’s worth continuing treatment anymore*,” referring to a user who did not accept her psychiatric diagnosis or, in the professionals’ terms, was “*lacking insight into her illness.*” The psychologist added that she believed it was a psychosocial case, and therefore the modality at that center was not appropriate (Fieldnotes, July 9, 2018).

This differentiation between “clinical cases” and “psychosocial cases” had a direct impact on the treatment options available within the facility. It seemed that the complexity justifying entry into secondary mental health care dismissed psychosocial variables, thereby reducing COSAM’s responses to pharmacological alternatives aimed at alleviating or diminishing clinical symptoms, or, alternatively, to hospitalizations when symptoms did not respond to medication. In this way, we begin to see how the psychosocial complexity, which is highly prevalent among users with mental disorders, was delegated to the sphere of primary care. This internalized a perspective within secondary-level services that dissociated the need for intervention in community and psychosocial problems, factors that sustain and even perpetuate symptomatology.

The tension arising from the coexistence of both models permeated treatment, since the strong presence of the biomedical approach contributed to a naturalization of biological explanations of behavior, which in turn reinforced prejudice and discrimination.^19^ This reflects the biologicist nature of biomedicine, where the subject is addressed from a reductionist perspective that strips them of their social and historical context,^13^^,^^14^ thereby overvaluing biological explanations of suffering at the expense of other possibilities for addressing it.

At the same time, this tension between community-oriented and biomedical perspectives was facilitated by the gap between institutional guidelines, which promoted a more community-based approach, and the actual resources available to implement it, as one professional noted:

*I believe that the macro policies in the field of mental health are not on the wrong track, no doubt, but they don’t match reality, you know? So manuals arrive here, distilled* [laughs], *distilled from the Ministry, and the truth is that you see things that don’t correspond either to the population, or to the reality of the population, or to the available resources, and therefore, neither to the number of staff - because resources, staff are part of the resources. So there are very few resources, really very few resources. So the ideas are good, but most of them just stay there*. (Interview with psychologist, September 7, 2018)

Institutional guidelines for community-based work in mental health are valued but perceived as impracticable, mainly due to their inadequacy to actual conditions and, above all, to the availability of professional resources. In this context, biomedical approaches in clinical practice may be prioritized, as they require fewer human resources for ambulatory implementation, leaving health teams in a constant tension between discourse and professional practice. In short, the biomedical model becomes evident primarily in how interventions are understood and how their impact is measured. This perspective frames the success of an intervention through variables tied to behavior that signal symptom reduction, while sidelining the social dimensions of recovery and emphasizing the power attributed to medication in producing improvement.^32^ This model shapes how professionals refer to users and how they act toward them. In this sense, diagnostic labels emerge as the undisputed guide for health care, with people being equated with such labels,^33^ producing a reductionist vision of the user.^34^^,^^35^ The label enables the embodiment of the role of “patient,” which is also reinforced by institutional organization and structure, as we will see below.

### The institutional praxis of the biomedical model of care

The model of care employed shapes the treatment trajectories of users and prescribes ways of thinking about and treating them that can convey stigma. Users must first assume the role of “patient” and recognize themselves as “ill” in accordance with the clinical assessments defined by the professional team, while adhering to the treatment prescribed on the basis of diagnosis.

Mental health units have had a dedicated space within the hospital premises since 2010. These facilities were conceived to function both as community mental health centers (COSAMs), providing outpatient services, and as day hospitals. The buildings remain in very good condition and feature a modern architectural style, creating the impression of delivering up-to-date, high-quality medical care. These sites include rooms designed for workshops and meetings (Field notes, June 12, 2018). While the facilities are well maintained and provide a pleasant setting for care, the rigid hierarchies embedded in the physician-patient relationship become visible in the clinical space.

For clinical meetings, a table is placed in the center, with the medical team sitting together in a row, and across from them a chair where the user sits to present their ailments and symptoms. Some professionals recognize how this physical arrangement of space may negatively impact users, as one psychiatrist acknowledged in an interview:

*…what can affect them a bit more is that - suddenly arriving and finding themselves - if they haven’t been told beforehand - with ten people, an audience of people where they’re alone there, or at most with one companion, and where they’ll have to present their case, and themselves, essentially, to a team of ten. But I’d say that if they haven’t been informed, it can be quite an impact. If they have been informed, it’s much less so.* (Interview with psychiatrist, October 23, 2018)

At the same time, when asked in a semi-structured interview about his views on clinical meetings, the occupational therapist at the COSAM remarked: “*I also find it kind of invasive, going in there and having them all sitting in front while the user is alone, telling their stuff… plus, it could be different somehow… maybe a round table, or something more friendly*.” (Interview with occupational therapist, October 7, 2018)

We note that both the ways in which space is organized and the infrastructure of the facility contribute to constructing the role of the “patient” in mental health care, as they generate differentiations in the uses of spaces. This is evident, for example, in the mental health center’s pharmacy, which various users jokingly refer to as *“the pharmacy for the crazy.”* It becomes a recognizable reference point for those who circulate through the facility. While the existence of a dedicated pharmacy within the center is perceived as a valuable resource and a highly positive feature -given the convenience of easy access for users- it also generates, at a social level, a differentiation between general health care and mental health care.

This leads us to consider how material and spatial arrangements shape the ways in which mental health users are perceived by their surroundings. In this case, they are separated from the hospital’s general pharmacy and relegated to a distinct social space, widely recognized as one exclusively for “the crazy.” Along similar lines, the space of the clinical meeting mentioned above constitutes another fundamental interactional setting, where, from the very moment the “patient” enters, hierarchical and role-based differences are underscored in the dynamics of clinical interaction, reinforcing and legitimizing processes of labeling.

Moreover, the tensions within institutional praxis are not only affected by infrastructure and equipment, but also by the modes of administration and funding of the centers, as one of the professionals interviewed noted:

*…in reality, there are very few resources* […] *because, for example, there should be many more protected residences here - many more! - and there are no protected residences that belong to the Ministry; they’re all private protected residences, and since they’re private, they always expect to take a cut. So there have been patients who had to be removed because they weren’t being properly cared for - despite paying quite a good amount. I think there should be more protected residences; but for that, you also need more public service staff who can work in those protected residences, you see? That’s just to give you an example.* (Interview with psychologist, September 7, 2018)

In Chile, the administration and financing of mental health services operate through a mixed system, concentrating care in public health centers while also relying on private entities that receive public funding through competitive bidding. These bureaucratic and institutionalized arrangements impact users’ experiences and care trajectories, as highlighted by health professionals, influencing the possibilities for adequate support. In this regard, Jenkins,^36^ analyzing the situation in the United States, accounts for the effects of budgetary and administrative changes in mental health centers on treatment continuity and discontinuity, ultimately affecting recovery outcomes.

### The mental health care trajectory as the institutionalization of users’ identities and its effects on health professionals’ practices

At this level, we focus directly on the stereotypes, prejudices, and stigmatizing behaviors exercised by professionals toward users, which contribute to the institutionalization of their identity, almost entirely associated with the diagnosis assigned to the individual. This, in turn, complicates the intervention and recovery processes for mental health service users.

Moreover, the implementation of treatment centered primarily on one aspect of the user - namely, pharmacological intervention - fosters the “chronification of patients” in mental health, understood as the creation of prolonged dependence on mental health services resulting from limited interventions that fail to address the complexity of the user’s social context.^21^ Chronification is associated with a pessimistic perception regarding the users’ potential for recovery.^37^ This perspective shapes the intervention options offered to individuals and diminishes their own hope for progress, ultimately impacting their recovery. The challenges in intervention and the limited achievements are perceived by staff as exhausting, leading them to develop various coping strategies to maintain their own well-being.

#### Processes of institutionalization of users’ identities in the clinical trajectory

The institutionalization of identity occurs primarily through the enactment of the “patient” role linked to the psychiatric diagnosis, which, as mentioned above, requires the user to assume and demonstrate “insight into their illness.”

During clinical meetings, users generally serve as narrators of their experiences, symptoms, and psychological-psychiatric conditions before the health care team, which positions them mainly as recipients of professional recommendations and conclusions. However, at times they asked about the effects of medications, and professionals typically responded in ways that either deflected the concern raised or reinforced the importance of taking their medications, emphasizing the benefits these would provide. For instance, in the clinical meeting on August 6, 2018, a psychiatrist responded to a user who asked about difficulties sleeping by telling her that it could be corrected with a medication. She replied that she believed the medications might not help or might even harm her, given that she was taking so many. The psychiatrist responded, *“It’s only two pills, at least in terms of mental health, that’s what it is,”* effectively closing the interaction without allowing the user space to follow up or negotiate the treatment.

In this context, during an interview, I explicitly asked the psychiatrist about patients’ attitudes as recipients of pharmacological prescriptions:

*Uh, let’s see, I think it depends on the patient. You must have noticed that there are patients who don’t have much awareness of what a treatment means - of the doses, for instance. That’s why this pillbox system is implemented, right? It’s managed by the occupational therapist together with the family members and, sometimes, with the patient themselves. Now, in psychiatry we have a small, quote-unquote, disadvantage: polypharmacy is kind of the rule - it’s very rare for a patient to be on just one medication. And that’s not because we want to solve everything with medication, but simply because that’s how the specialty works. So, yes, sometimes some patients may feel somewhat overwhelmed, you know, by having to take, I don’t know, three or four medications a day at different times. But that’s a kind of - let’s call it - a downside, or a characteristic of the specialty; I’d say polypharmacy is almost the rule, and monotherapy is the exception - in other words, a patient who’s on just one drug is the exception.* (Interview with psychiatrist, October 23, 2018)

This minimization of the user’s role is not only evident in pharmacological prescription but permeates the entire care trajectory,^38^ affecting treatment options and recovery potential. Users face restrictions in their participation, which undermines their rights in health care.^39^^,^^40^ Professionals’ perceptions of users are often shaped by prejudices regarding their capacities; users are seen as incapable and therefore in need of protection and care, fostering paternalistic and/or overly compassionate attitudes.^41^^,^^42^

These practices contrast with the theoretical principles of the community-based mental health care model, which values user and family participation in treatment, recognizing the knowledge of others within a horizontal and dignifying relationship.^43^ Conversely, the biomedical approach subordinates the user’s perspective to that of the health care team, who are regarded as the experts. This reinforces hierarchies within a power-differentiated framework^44^^)^ and leaves little room for negotiating treatment.

It is common, during clinical meetings, to hear professionals emphasize the need for users to possess “insight into their illness,” meaning that once diagnosed, the individual should adjust their behavior and self-conception based on the mental health label assigned by professional judgment, which is primarily grounded in statistical manuals of diseases and health problems, such as the ICD-10. When a person lacks “insight into illness,” the professional team seeks to foster its emergence, considering it central to successful intervention. An example of this is reflected in a clinical meeting exchange, where the social worker recalls how the user appeared previously and states:

*Now she’s seen more as a patient; before, she would just come by for specific things, to ask questions here and there. The psychologist adds, “there was no awareness of illness,” and as a team they value the work done by the professionals at the day hospital on this issue, both with the user and with her family.* (Clinical meeting, July 23, 2018)

*Another user, during a clinical meeting, asks whether the anxiety she’s feeling might be a side effect of a medication. The psychiatrist tells her, “the medication is helping you, not harming you.” Immediately afterward, the doctor asks about her children and whether she feels capable of taking care of them. Closing the interview and thanking her for coming, he tells her, “let’s do what we’re told,” referring to the act of taking the prescribed medication.* (Field notes, June 19, 2018)

In summary, it can be observed that, although the theoretical framework emphasizes a community-based approach, in practice there is a strong tendency to delegate authority to the professional. This fosters a paternalistic perspective that undermines the user’s autonomy and capacities, leaving them dependent on medical directives, as evidenced in various clinical meeting situations.

### The impact of mental health work on professionals and their coping strategies

The complexity of users’ life situations - which often exceed the scope of pharmacological interventions and require a psychosocial approach - combined with, on the one hand, the limited availability of psychosocial interventions and, on the other hand, the constant tension between the model promoted discursively and that imposed structurally and in daily practice, contributes to professionals developing a limited view of themselves in their roles and their capacity for intervention. This has repercussions for professional practice, facilitating emotional and cognitive exhaustion. Mental health work generates a range of feelings among professionals, with sadness and anger being predominant. In the words of one professional, what she finds most exhausting is:

*The cases we see are complex, there are all kinds of problems, and honestly, it makes me feel sad… anyone can develop a mental health condition. I have children; I don’t know what might happen to them in the future. Sometimes I’m just grateful that everything is fine in my family. It’s mostly that - working with people’s problems.* (Interview with social worker, August 10, 2018)

Anger, on the other hand, seems to be associated with users who do not follow the team’s requirements, which generates weariness, while sadness arises in cases where users’ life circumstances are unfavorable. In one of the team meetings, the psychiatrist reflects on what happens when dealing with the case of a user (Ana), for whom the team is considering once again “setting boundaries,” that is, establishing, marking, or reinforcing the limits and expectations associated with being a mental health patient. To this end, she is compared with another user (Berta), who had also been scheduled for that same day and who attends with similar irregularity. However, despite this, her case has not led the team to reconsider the treatment or the “framework” to be applied. In this context, the physician asks the team:

*“What’s the difference between Berta and Ana? That Berta makes us feel pity and Ana makes us feel anger - we have to manage that; it’s part of the burnout in our work.” In response, the colleagues share this view, and then the psychiatrist sighs, saying: “We’re exhausted by this case… just by talking about it.”* (Field notes, June 25, 2018)

There are instances in which cases move professionals and affect them emotionally, particularly when a user’s experience resonates with the professional on a more personal level. This was the case of a user, approximately 50 years old, who attended accompanied by her partner. After the interview, the team commented on the impact of the user’s account. One of the psychologists on the team stated:

*“…personally, it really affected me to feel her suffering,” and the doctor comments, “she’s truly suffering, huh,” to which another psychologist participating in the meeting adds, “oh, I feel sorry for him, it reminded me of an uncle who’s like that , so in love with his wife, accompanying her and suffering because she’s not well.” Then, a social worker asks, “What do we do?” And the psychiatrist, who joined halfway through the interview, comments that he believes the diagnosis is indeed schizophrenia.* (Field notes, August 21, 2018)

The effects of sadness on professionals also depend on the coping strategies each individual possesses and how they implement them. Some evade the feeling, focusing instead on practical matters and defining specific actions regarding the user, while others are moved by this emotion and become involved beyond what is typically expected in their role, as one professional noted:

*“I feel sad - you see so many problems that sometimes it really affects me, and that makes me try to help beyond what would normally be expected. For example, if I see a patient who’s struggling, and I realize they don’t have a job and have small children, I go and buy them some things myself, but then I tell them that I arranged it through others and that the things were sent to them, you know what I mean? Or sometimes I’ve gone to visit them at home, just using my own car - I mean, what does it cost me? And it’s not so much about being charitable; it’s more that if I can help, why wouldn’t I?”* (Interview with social worker, August 10, 2018)

This feeling can lead professionals to adopt positive attitudes toward users,^45^^,^^46^ which on some occasions manifest as excessive compassion.^42^ Furthermore, cognitive exhaustion refers both to the fatigue generated by the administrative workload inherent to health work and to the intellectual effort required for the constant clinical analysis of the cases attended. In addition, the fact that schedules are consistently filled due to the large number of users to be seen forces multiple individual consultations per day, without sufficient space to decompress and take breaks between appointments. This is perceived as being “atomized” and is conceptualized as unpleasant.

Another aspect included in this type of exhaustion is the effect produced by user behaviors that are categorized as difficult to manage by professionals, such as the “setting boundaries” cases mentioned previously. It is common for professionals to remark, *“it left me exhausted,”* as noted by the psychiatrist, referring to a user who was excessively talkative, making it difficult to manage the flow of conversation during the interview (Field notes, June 19, 2018). From this, we infer that what exhausts professionals is anything that prevents the usual flow of the interview dynamic and demands that they adjust their actions to the situation, requiring greater effort. This can also lead to extended meeting sessions, as such situations are complex to address.

Exhaustion is also generated by the diverse skills required when working in mental health. This is a performance area that demands close attention to what is done and how it is done, requiring both technical and socio-emotional competencies. The work is perceived as challenging due to its demanding nature, necessitating capacities beyond the ordinary. Moreover, this work requires an integrative approach, in the sense of balancing technical expertise and personal engagement. In other words, it requires attention to both the environment and oneself, which can make the work particularly draining, as clearly reflected in the words of one professional during a semi-structured interview:

*“Besides having to stay focused on the technical aspects - formulating the diagnostic hypothesis, outlining the treatment plan - you also have to be very attentive to yourself, right? To what’s happening to you… because patients generate emotions, they generate what’s known as countertransference. So it’s a double task - a double task that also happens with other patients! You also have to keep checking yourself: ‘Okay, this patient has schizophrenia; am I really getting into their clinical case? Am I managing to get to the core of it?’ if that’s not happening. But it’s much less difficult than with other cases, for instance, personality disorders, where you have to draw on every therapeutic tool you have to maintain the neutrality that’s absolutely necessary for success in these cases. Now, depending on one’s experience, age, and how long one’s been practicing, this tends to become more automatic, right? But when you suddenly feel stuck or sense that something isn’t working, you have to stop for a moment and start asking yourself: ‘Okay, what’s happening to me? What’s the countertransference?’”* (Interview with psychiatrist, August 23, 2018)

Exhaustion resulting from working in mental health, particularly emotional exhaustion, is recognized by professionals as an “occupational hazard,” that is, as part of the responsibility they must assume as health care workers. Emotional exhaustion is considered an inevitable effect of constantly witnessing and/or connecting with adverse symptoms, behaviors, and psychosocial conditions that reflect the suffering of the individuals they serve.

To address this, certain strategies have been implemented to allow staff to decompress, creating what they call “self-care” sessions. These occur monthly and typically involve dedicating half a day to a joint extracurricular activity, such as going out to eat at an external venue. These sessions are funded through a mixed scheme, with contributions from both the institution and the staff, aiming to foster relaxed interactions and provide shared moments of leisure.

Additionally, in response to the exhaustion described, another factor that professionals recognize and value as protective is the presence of interaction spaces among colleagues within the work setting, such as the joint analyses carried out during clinical meetings. These work-related instances are also experienced as opportunities for “self-care,” allowing professionals to discuss their experiences with particular users and, at times, to employ humor among colleagues as a way of limiting the emotional burden of their work.

However, it is observed that for some professionals, this emotional exhaustion, being continuous and routine, can lead, as a defense mechanism, to a disconnection from the emotions and suffering of the users. This may result, in some cases, in the standardization and automation of care and intervention processes, as the behaviors, conduct, and symptoms of different users tend to be perceived as equivalent. This limits the possibility of engaging in a phenomenological understanding of each person’s unique experience.

Furthermore, this dynamic can desensitize professionals, who may focus more on symptoms and diagnoses than on the individual experience of the person. Consequently, the risk of dehumanizing care processes and perpetuating stigma increases, reinforcing stereotypes about what a mental health service user can and should be like.

## Discussion

The findings presented in this study allow for reflection on the tensions and contradictions that emerge between the biomedical approach and community mental health perspectives. In particular, they highlight the difficulties and points of convergence between an institutional discourse that does not necessarily align with mental health care practice.^20^^,^^21^ We observed that the implementation of a community-based mental health model, while part of an effort to adapt mental health services to sociocultural inequalities and diversity,^47^ has not yet been fully consolidated.^48^

This is sustained by the opposition between the biomedical care model and the community model. This tension permeates not only the treatment of individuals with mental disorders but also affects the labor, medical, and care practices carried out by mental health professionals.^20^^,^^48^ Declaratively, care is guided by a community-based mental health model, whereas in practice, the biomedical perspective predominates, forming part of the hegemonic medical model,^13^^,^^14^ which, in the mental health domain, bases its explanatory framework on disease and neurochemical imbalances over social determinants that may influence mental health conditions.^49^ This naturalizes biological explanations of behavior and contributes to the reproduction of prejudice toward individuals diagnosed with mental health conditions.^19^

The primacy of the biomedical over the community perspective^50^ is reflected in the tension between clinical versus psychosocial approaches, the latter being associated with the community model. Although valued by mental health professionals, this approach is perceived as difficult to implement in light of the social and economic realities of the population served, as well as the limitations in financial and professional resources available to care services. This can result in the social context of the user being considered relevant mainly for determining pharmacological treatment; however, the psychosocial dimension is not integrated as an essential aspect of the individual, but rather regarded as secondary within the therapeutic exercise.^49^

Furthermore, the difficulties or contradictions identified are part of structural inequalities that subject mental health professionals to excessive workloads.^47^ As highlighted in this study, working in mental health entails persistent wear and tear for those practicing it, a phenomenon widely documented in the literature.^51^ The so-called burnout syndrome, understood as emotional exhaustion and professional fatigue, is particularly pronounced among mental health staff.^52^ However, rather than an individual trait, this exhaustion is shaped by the conditions of practice itself: attending to individuals with complex, difficult-to-resolve problems, where progress is perceived as slow or almost imperceptible, generating a sense of limitation and helplessness within the teams.^51^^,^^53^

Within this framework, users who resist institutional logics - those who do not conform to the established frameworks or biomedical prescriptions - further intensify the daily labor. Far from being mere “difficult patients,” they serve as a reminder that system norms cannot always accommodate the diversity of life trajectories, which simultaneously increases the effort required from the teams and exposes these users to greater risks of being stigmatized.^54^

Mental health staff exhaustion is also fueled by constant exposure to human suffering in a context of high technical and personal demands. Although recognized as specialists, many perceive that they lack the necessary tools to sustain this work, reflecting the tension between societal expectations placed on their role and the actual possibilities offered by the system. This tension is exacerbated in contexts such as Chile, where mental health has historically received limited resources: in 2021, only 1.7% of the national health budget was allocated to this area.^55^ This structural precarity translates into overwork, overloaded schedules, and impossible demands, reinforcing a cycle of wear and strain that has been documented in other contexts as well.^56^^,^^57^

It is important to emphasize that mental health professionals do not remain passive in the face of exhaustion but deploy various coping strategies. In this study, one of the most relevant was the use of social support within the work environment, understood as a means of managing stress that offers significant benefits to those who utilize it.^58^^,^^59^ Institutionalized spaces, such as “self-care” sessions or clinical meetings, acquire particular value in this regard: beyond their technical dimension, they serve as venues where professionals can socialize with peers, share experiences, and collectively process the emotions elicited by daily work. These moments, by facilitating the circulation of speech and empathy among colleagues, function as a buffer and relief against the pressures of day-to-day work.^60^

Another strategy that emerges strongly is desensitization to users’ problems.^61^^,^^62^ While this allows professionals to create a certain emotional distance that mitigates the impact of others’ suffering, its effects directly influence the therapeutic relationship. This coping mechanism can erode the quality of the professional-user relationship, affecting treatment adherence, satisfaction with care, and ultimately the recovery process itself.^63^ The cycle is closed when mental health staff frustration, stemming from perceived stagnation or chronicity in some users, fosters new forms of stigmatization: users are seen as responsible for their lack of progress, which reinforces negative attitudes toward them and further strains the recovery trajectory, an area that warrants continued investigation.

## Conclusions

Stigma toward individuals with psychiatric diagnoses by health personnel is anchored in the trajectory of mental health care. This trajectory is intersected by macrosocial factors linked to the tension between two models of care: the community-based mental health model and the biomedical model. The former is promoted by the country’s mental health policies,^7^ while the latter is embedded in routine professional practices. Social determinants of mental health are scarcely addressed in daily practice, leaving psychosocial aspects subordinated to biomedical concerns. This model frames and addresses suffering in a way that prioritizes technical knowledge associated with diagnosis and medication prescription. Consequently, the expertise of other professionals is undervalued, and the participation of diagnosed individuals in their own care is reduced. Diminishing the user’s agency constitutes a form of stigmatization, as it reflects a view that undermines their capacities and potential, which can affect recovery and contribute to chronicity.

Furthermore, the biomedical model, by emphasizing diagnosis and pharmacological treatment, prioritizes the need for users to possess “insight into illness,” understood as accepting their identity as “patients” and following medical prescriptions without question. The pressures exerted by the care team to adhere to treatment can therefore be considered coercive, representing one of the ways stigma is enacted.^64^

Tensions between these models are also reflected in how the health system organizes and allocates resources. While a community-based mental health model is promoted in principle, professionals report that resources are insufficient for its implementation, whereas mental health staff are expected to meet standards focused on individual consultations. In carrying out their work, health personnel experience a split between these expectations and the realistic possibilities of compliance. This situation contributes to a constrained self-perception regarding their role and the scope of their interventions. Coupled with the inherent challenges of working with human suffering in a role that demands high technical and personal capacities, professional exhaustion appears inevitable. This, in turn, impacts the relationship with users, thereby facilitating stigmatization.^65^

Nevertheless, mental health personnel have developed coping strategies that help them manage these challenges, highlighting their active role in the process. Some strategies are personal, such as avoidance, while others are collective, centered on what teams refer to as “self-care.” This approach aims to alleviate everyday work-related tensions and strengthen trust among health personnel through activities generally of a recreational nature. Similarly, clinical meetings are not only technical in nature but also constitute a source of social support in the workplace. From this perspective, they are part of collective care strategies among staff. In a collaborative work environment, care cannot be an individual task but must - and in practice does - function as a collective endeavor. Caring for oneself implies trust, reciprocity, and mutual support, enabling staff to better face the challenges of demanding work.

In this study, stigma appears anchored less in individual stereotypes or attitudes and more in macro-, meso-, and microsocial factors shaped by the model of care and the structure of the health system. These factors impact the daily practices of professionals, particularly in their conceptualization of what happens to people with mental health diagnoses and how to address it. Therefore, reducing stigmatization requires examining the system in which it arises and implementing interventions that incorporate internal modifications. As long as efforts focus solely on improving the attitudes of mental health personnel toward users while neglecting the social and political framework that shapes these attitudes and stigmatizing behaviors, changes will remain limited. Moreover, it is essential to consider the repercussions for staff working in mental health: the challenges inherent to this labor must be addressed not only to prevent professional burnout but also to mitigate its effects on interpersonal interactions with users, which are a direct consequence of occupational fatigue.
